# Supplementary material for: Behavior control of membrane-less protein liquid condensates with metal ion-induced phase separation
Source: Nat Commun. 2020 Nov 3;11:5554. doi: 10.1038/s41467-020-19391-8 (PMC7642319; doi:10.1038/s41467-020-19391-8)
Supplement: Supplementary file 1 — Supplementary Information [file 41467_2020_19391_MOESM1_ESM.pdf]

## Supplementary Information

# Behavior control of membrane-less protein liquid condensates with metal ion-induced phase separation

**Kibeom Hong, Daesun Song, and Yongwon Jung***

*Department of Chemistry, Korea Advanced Institute of Science and Technology, Daejeon 305-701, Korea. E-mail: ywjung@kaist.ac.kr*

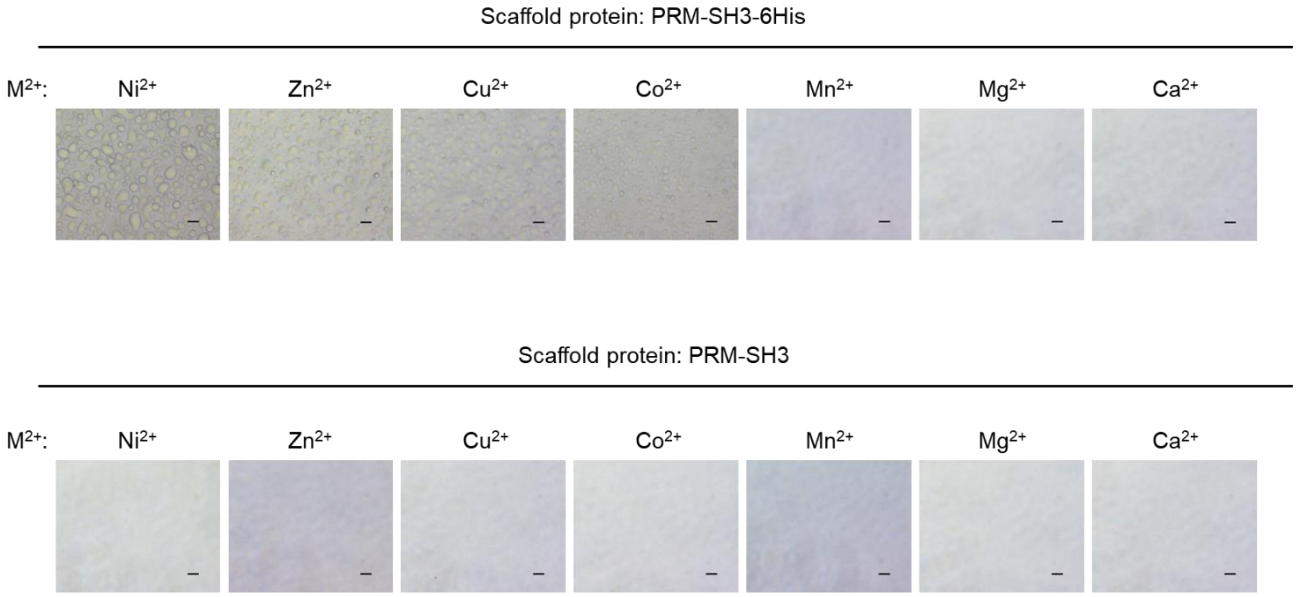


**Supplementary Figure 1** Protein phase separation of PRM-SH3-6His (top) or PRM-SH3 without 6His (bottom) by various divalent metal ions. 100 μM protein and 100 μM metal ion were mixed, and optical images were obtained after 30 min upon divalent metal ion addition. Scale bars: 10 μm.

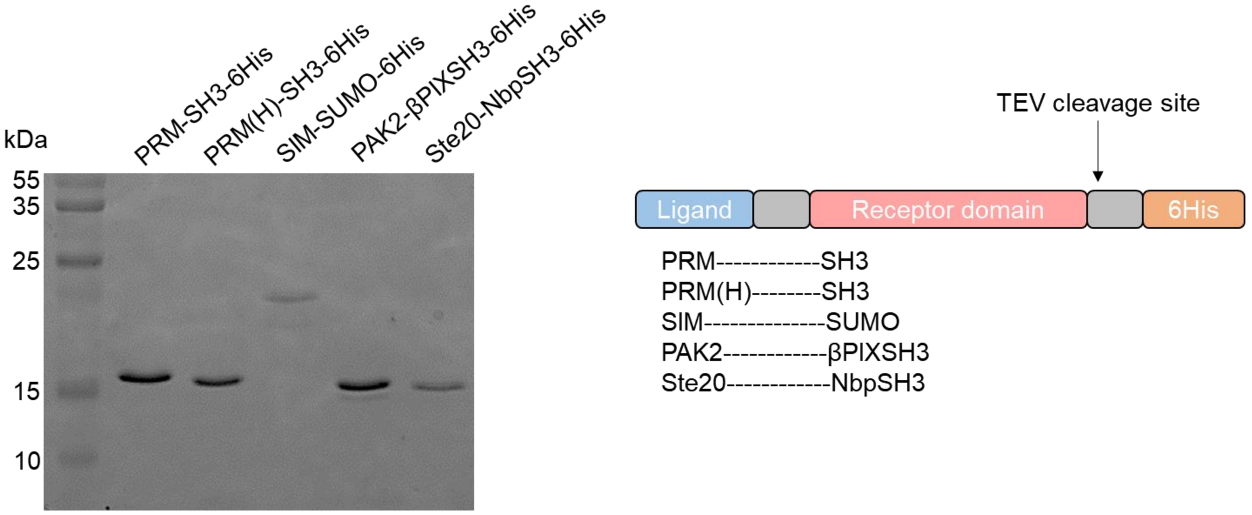


**Supplementary Figure 2** Ligand-receptor-6His scaffold proteins. A SDS-PAGE image and schematic description of scaffold proteins are shown.

a

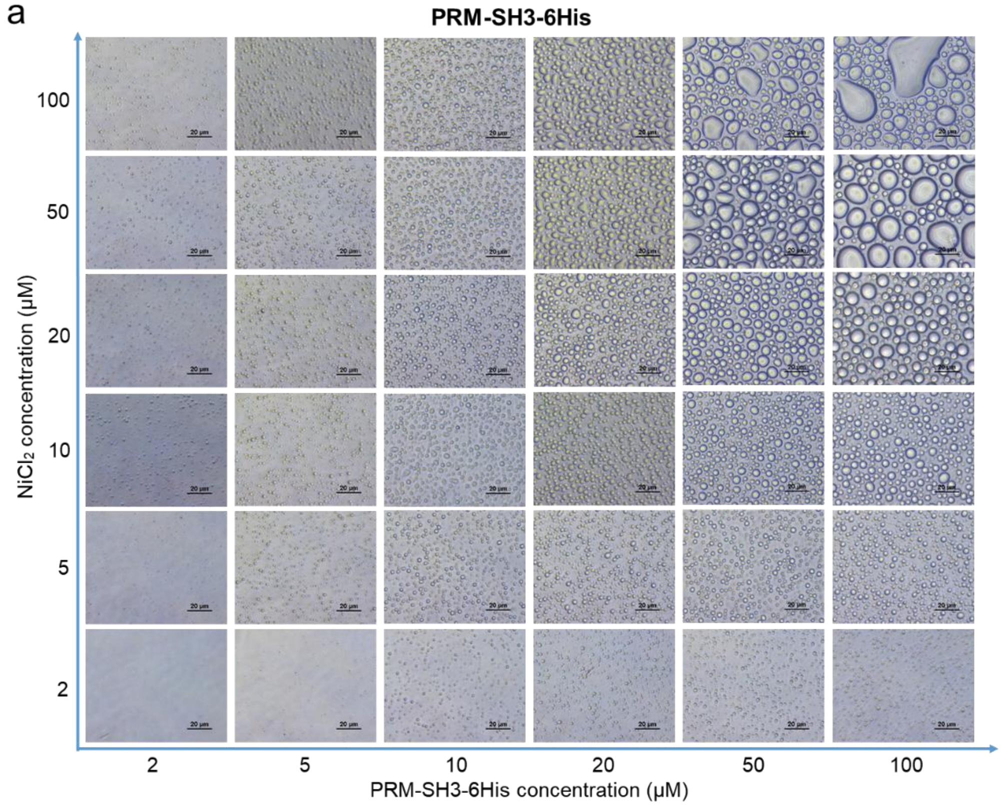


b

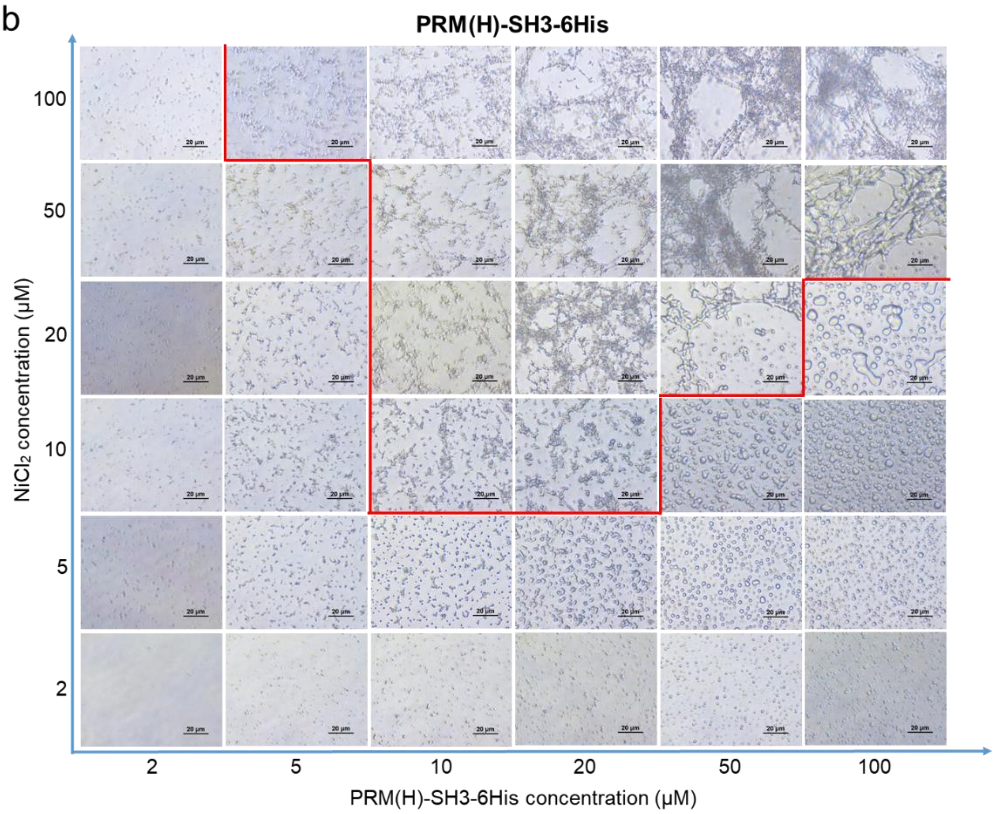


**Supplementary Figure 3** Optical images of phase separation diagrams of five scaffold proteins. (continue)

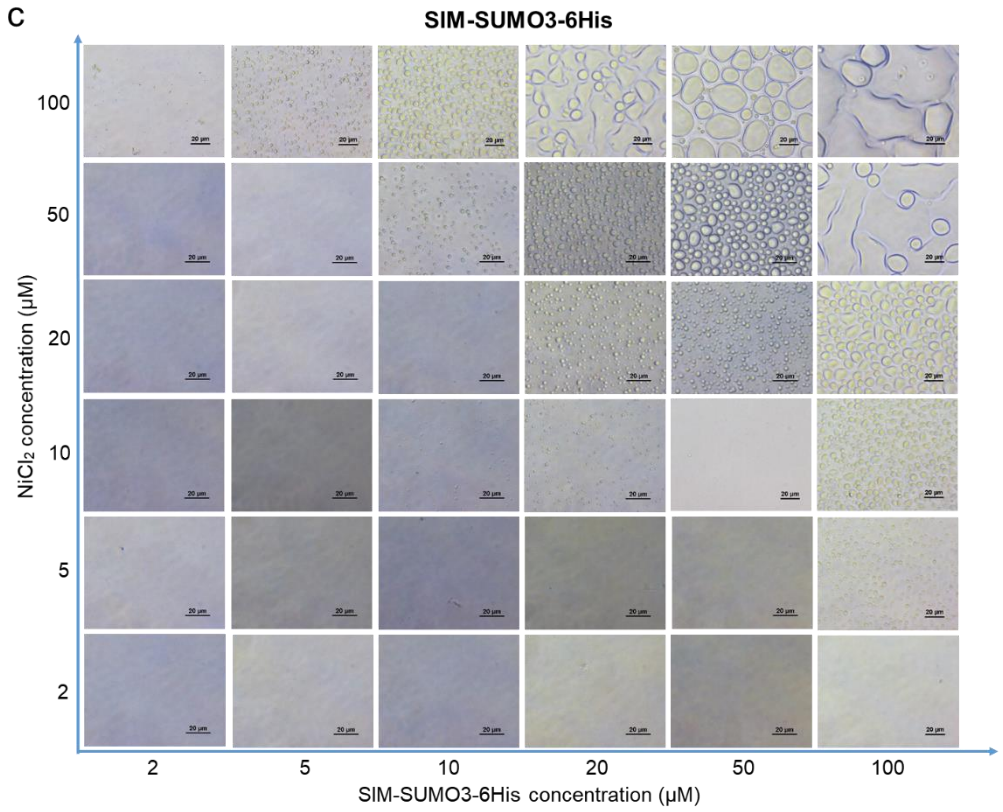


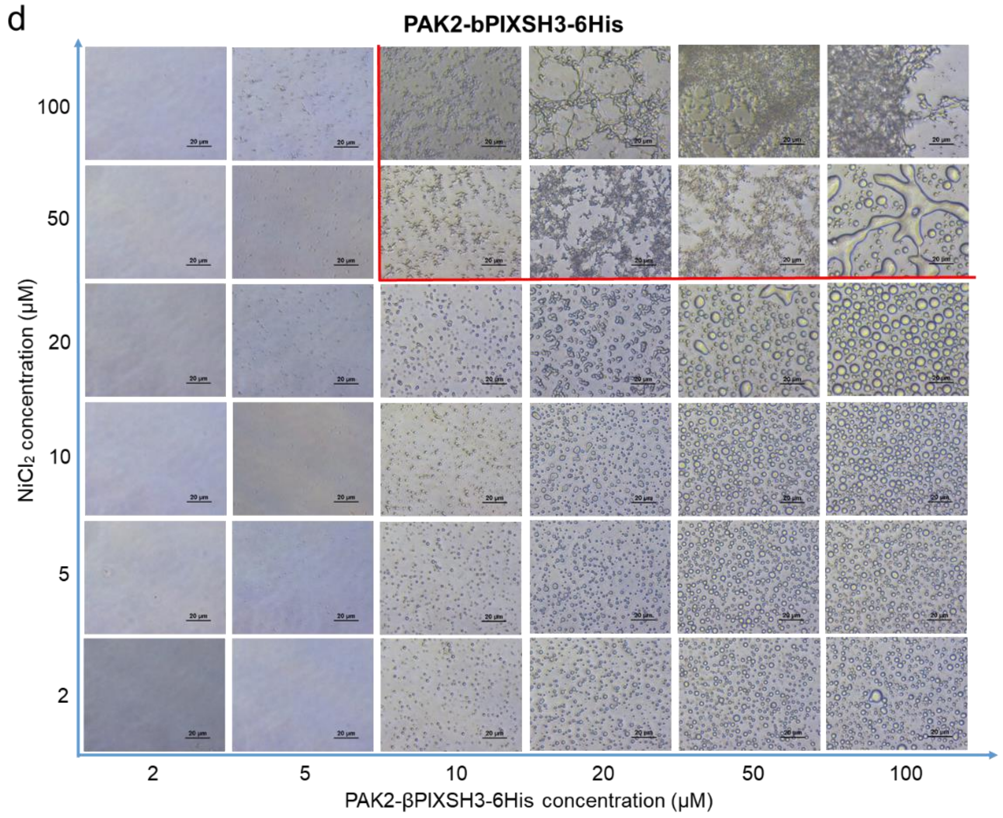


**Supplementary Figure 3** Optical images of phase separation diagrams of five scaffold proteins. (continue)

e

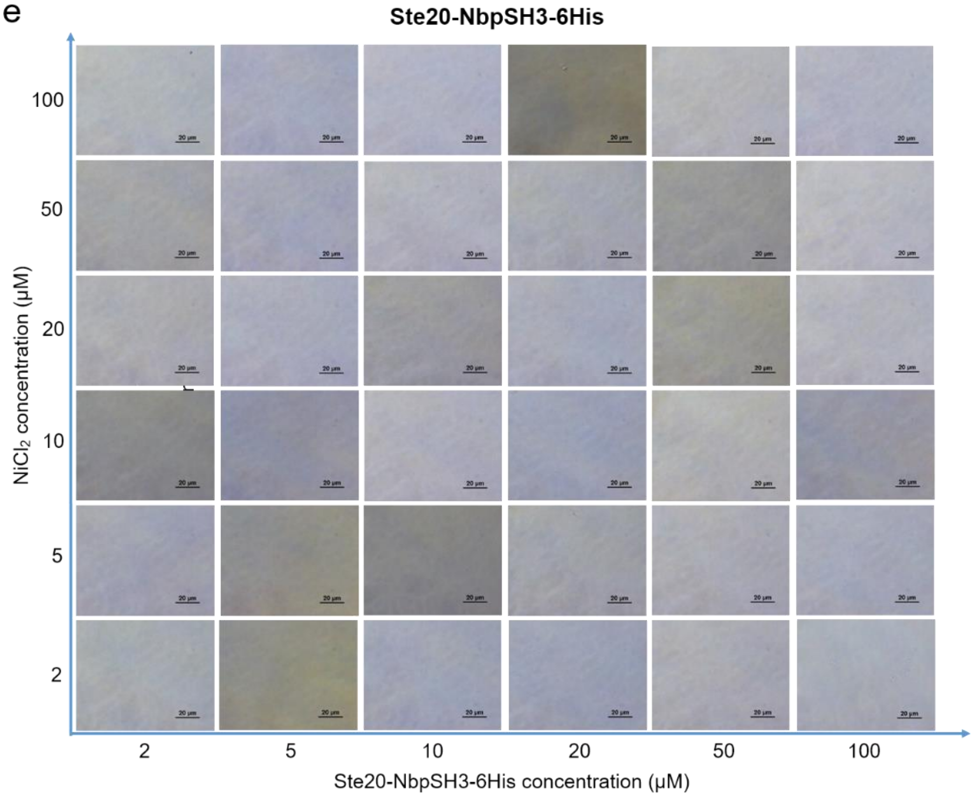


**Supplementary Figure 3** Optical images of phase separation diagrams of five scaffold proteins. Droplets with gel-like structures are indicated with red lines. Phase diagram images of Fig. 1e. Scale bars: 10 µm. Images were taken after 24 h upon $Ni^{2+}$ addition.

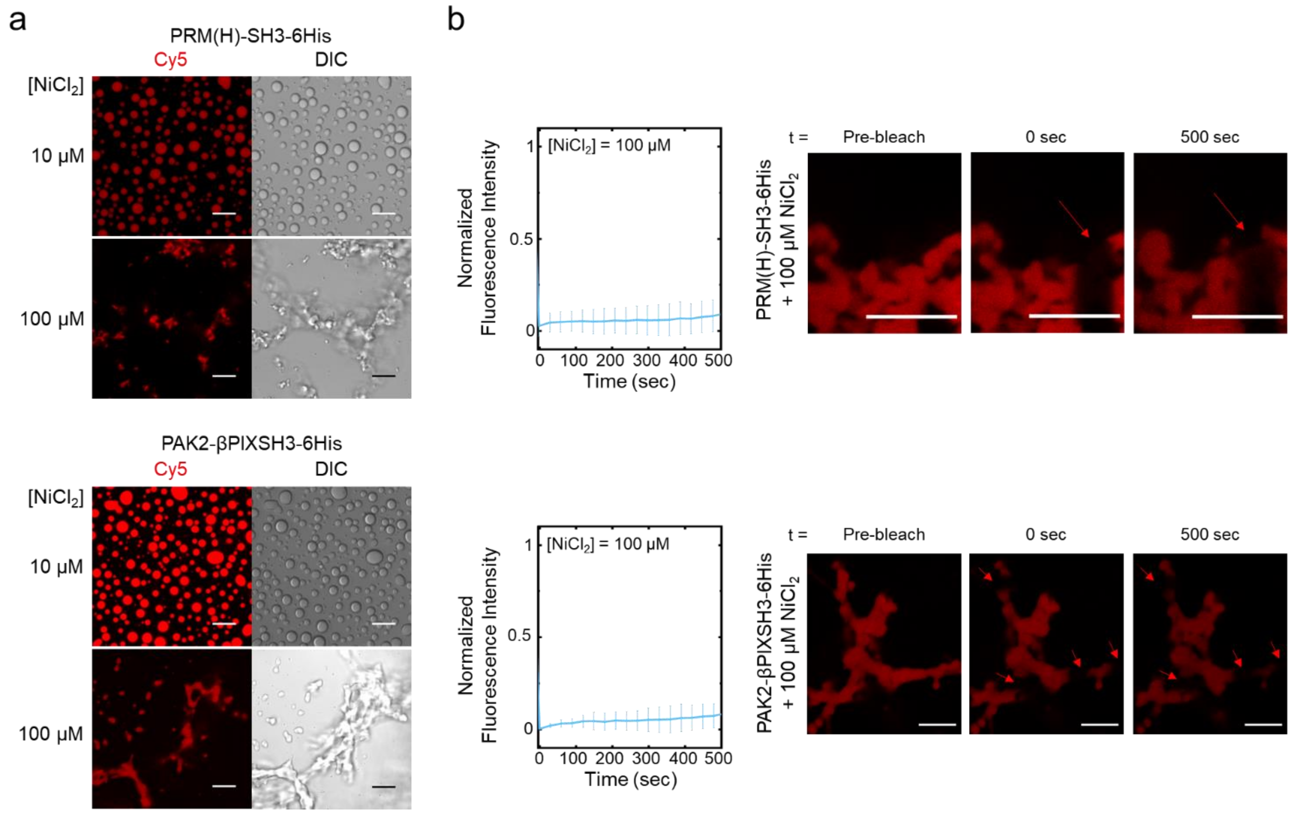


**Supplementary Figure 4** Confocal and FRAP analyses of PRM(H)-SH3-6His (top) and PAK2-bPIXSH3-6His (bottom) protein condensates at $[Ni^{2+}]$ = 10 µM or 100 µM. (a) fluorescence and optical images of protein condensates. (b) FRAP recovery profiles and images of protein condensates at $[Ni^{2+}]$ = 100 µM. S.D. from triplicate experiments with at least 7 condensates. Scale bars: 10 µm. Condensates were analyzed after 12 h at 25 ºC upon $Ni^{2+}$ addition.

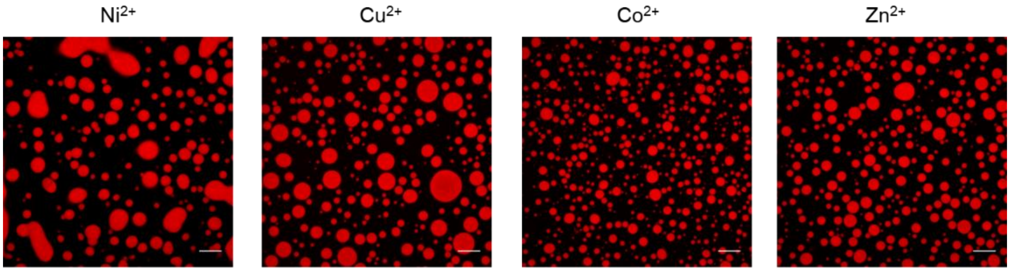


| Metal ion | P.C. | S.D. |
|---|---|---|
| $Ni^{2+}$ | 29.6 | 3.33 |
| $Zn^{2+}$ | 22.8 | 5.92 |
| $Co^{2+}$ | 14.0 | 4.70 |
| $Cu^{2+}$ | 13.2 | 5.26 |

**Supplementary Figure 5** Fluorescence images of protein condensates containing Cy5-PRM-SH3-6His with four different metal ions. Scale bars: 10 μm. Partition coefficients (PCs) of the scaffold protein are indicated in the table below. S.D. from triplicate experiments with at least 302 condensates. Condensates were analyzed after 1 h at 25 ºC upon $Ni^{2+}$ addition.

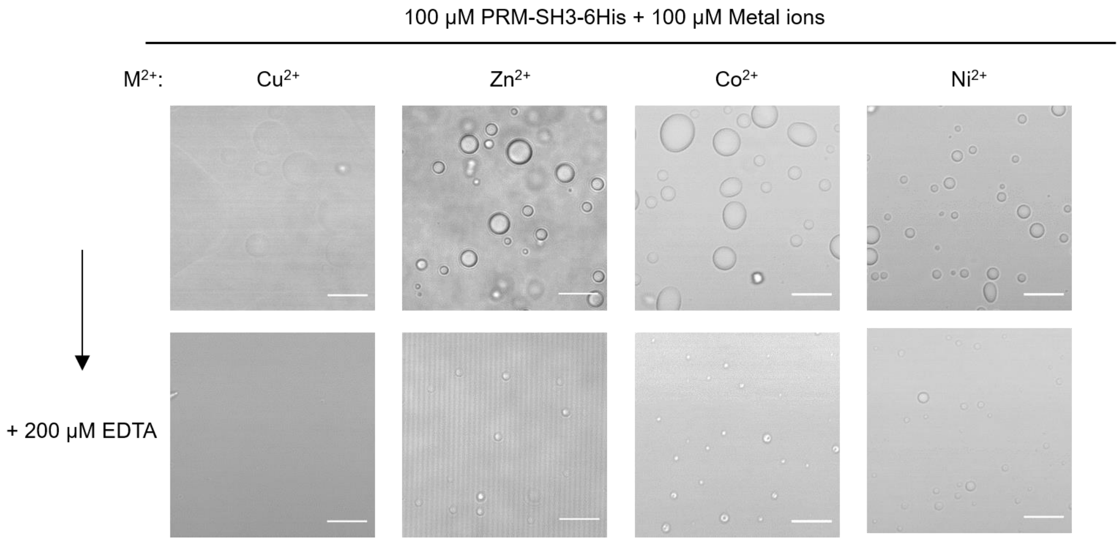


**Supplementary Figure 6** EDTA-mediated dissolution of protein condensates formed with four different metal ions. Condensates were formed for 60 min (top images) and treated with EDTA for 5 min (bottom images). Scale bars: 10 µm.

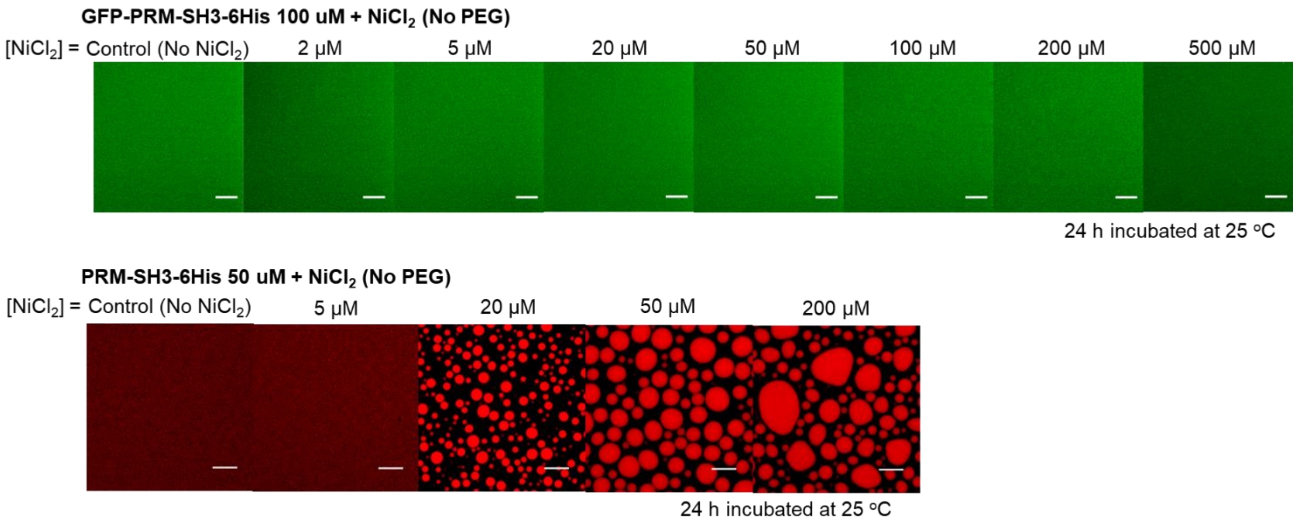


**Supplementary Figure 7** Fluorescence confocal microscopy images of 100 μM GFP-PRM-SH3-6His (top) and 50 μM PRM-SH3-6His (bottom) with varying $Ni^{2+}$ concentrations. Proteins and metal ions were mixed without PEG as conducted for DLS analyses. Scale bars: 10 μm

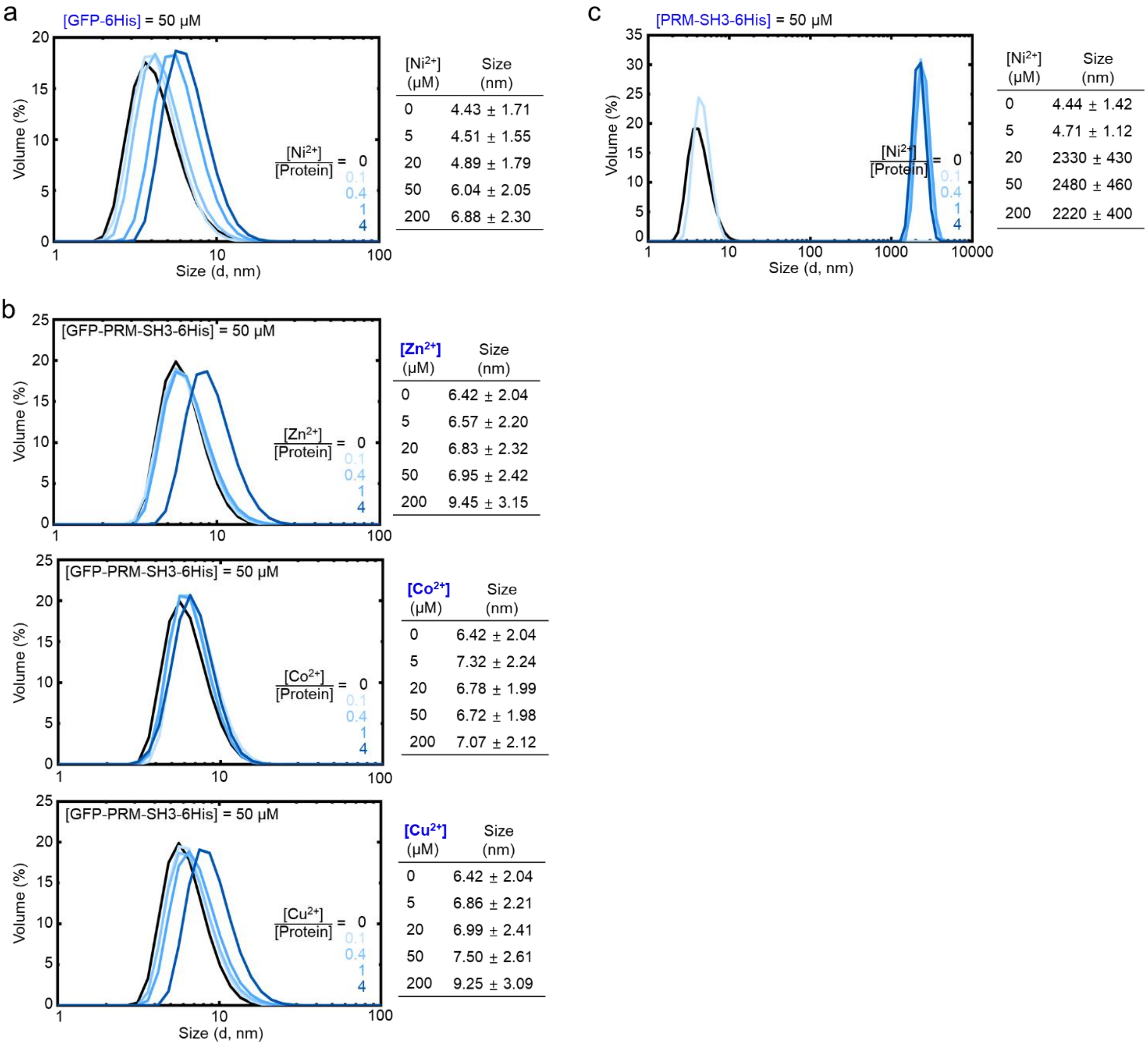


**Supplementary Figure 8** DLS analysis of scaffold protein clustering by metal ions. (a) DLS size distribution profiles of GFP-6His with varying ratios of $Ni^{2+}$. Average sizes at different $Ni^{2+}$ concentrations are indicated in the right table. (b) DLS size distribution profiles of GFP-PRM-SH3-6His with varying ratios of $Zn^{2+}$, $Co^{2+}$, and $Cu^{2+}$. (c) DLS size distribution profiles of PRM-SH3-6His with varying ratios of $Ni^{2+}$. S.D. n = 3.

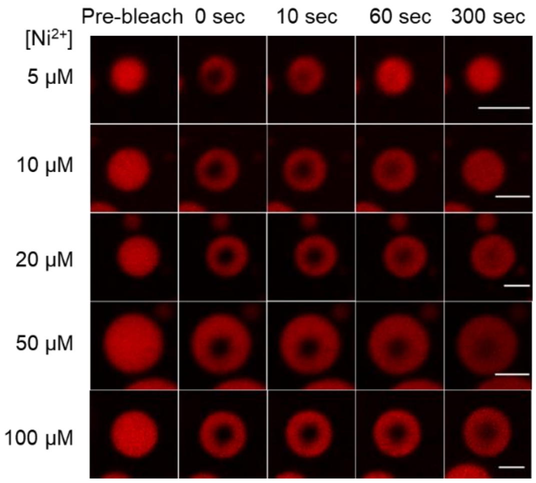


| $[Ni^{2+}]$ (μM) | Mobile Fraction (%) | S.D. | $t_{1/2}$ (sec) | S.D. |
|---|---|---|---|---|
| 100 | 36.4 | 21.6 | 145 | 82.2 |
| 50 | 43.8 | 9.11 | 103 | 19.6 |
| 20 | 63.7 | 7.66 | 78.3 | 18.5 |
| 10 | 83.8 | 5.08 | 40.6 | 7.27 |
| 5 | 95.9 | 5.50 | 15.6 | 5.52 |

Protein = 100 μM
60 min incubation
Selected droplets 50 ~ 10 $\mu m^2$

**Supplementary Figure 9** FRAP recovery images of 100 μM PRM-SH3-6His inside condensates with different $Ni^{2+}$ concentrations (Fig. 2e). S.D. from triplicate experiments with at least 18 condensates. Scale bars: 10 μm. Condensates were analyzed after 1 h at 25 ºC upon $Ni^{2+}$ addition.

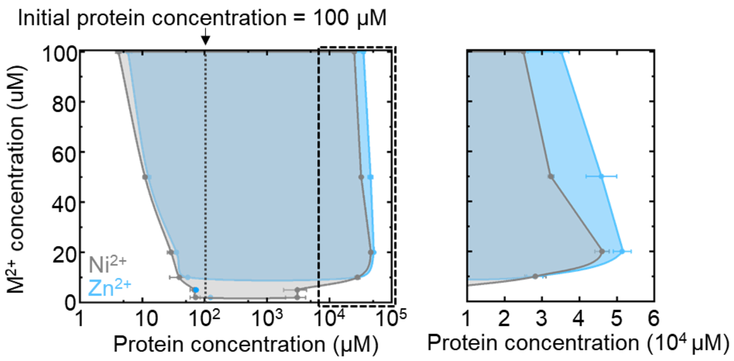


| [M2+] (μM) | Ni2+ | | Zn2+ | |
|---|---|---|---|---|
| | [Dilute] (μM) | [Dense] (μM) | [Dilute] (μM) | [Dense] (μM) |
| 2 | 71.5 ± 13.3 | 3060 ± 1080 | - | - |
| 5 | 68.1 ± 9.61 | 3070 ± 1230 | - | - |
| 10 | 39.3 ± 11.7 | 28200 ± 2820 | 53.6 ± 2.07 | 28000 ± 2160 |
| 20 | 28.9 ± 3.70 | 46000 ± 1910 | 35.3 ± 1.30 | 51400 ± 2360 |
| 50 | 11.0 ± 0.767 | 32300 ± 784 | 12.4 ± 1.24 | 45900 ± 4090 |
| 100 | 4.14 ± 0.417 | 25000 ± 1180 | 5.88 ± 0.86 | 35100 ± 2000 |

**Supplementary Figure 10** The phase diagrams of PRM-SH3-6His with dense and dilute phase protein concentrations as a function of metal ion ($M^{2+}$ = $Ni^{2+}$ or $Zn^{2+}$) and PRM-SH3-6His concentrations. Left arms exhibit dilute phase concentrations and right arms for dense phase concentrations of the scaffold (PRM-SH3-6His) protein. The magnified version of the right arms (dash box in the left diagram) is drawn (right diagram). S.D. n = 3. The $Ni^{2+}$ data are same as Fig. 2e, but included for better comparison with the $Zn^{2+}$ data. Condensates were analyzed after 24 h at 25 °C upon $Ni^{2+}$ addition.

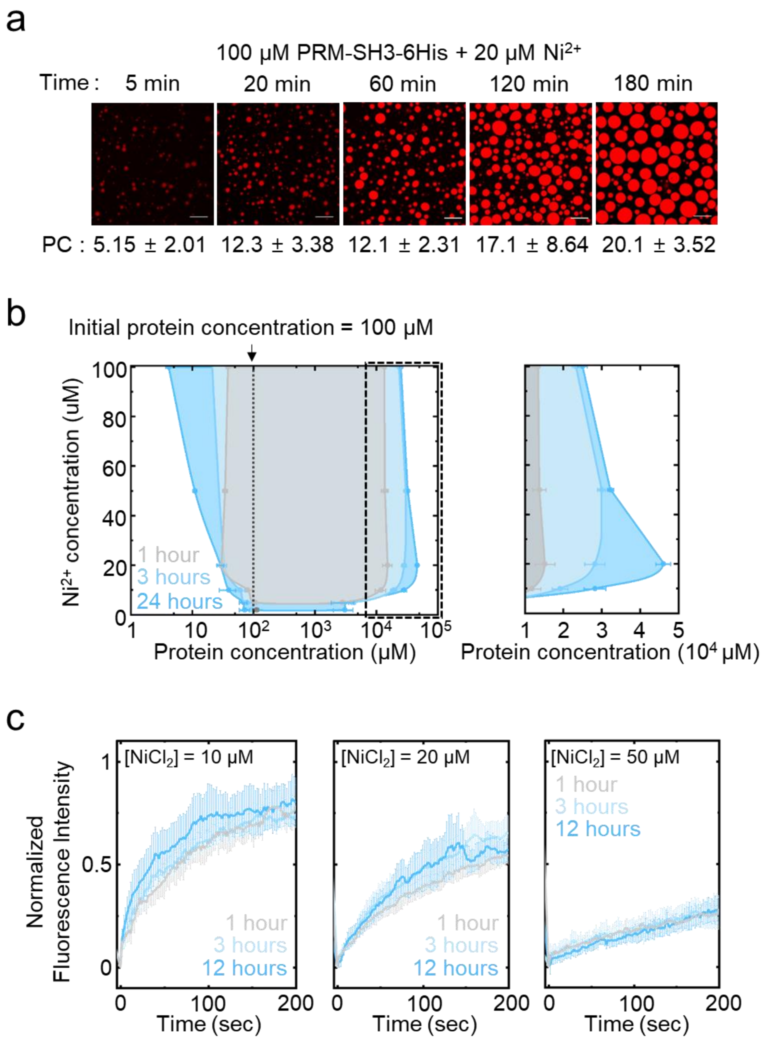


**Supplementary Figure 11** Incubation time-dependent changes of protein condensate properties (a) Fluorescence images of PRM-SH3-6His condensates with varying incubation time. Scaffold PC values are indicated below. Scale bars: 10 µm. (b) The phase diagrams of PRM-SH3-6His with dense and dilute phase protein concentrations at incubation times 1 h, 3 h, and 24 h. The 24 h data are same as Fig. 2e, but included for better comparison with the 1 h and 3 h data. (c) FRAP recovery profiles of PRM-SH3-6His with different incubation time (1 h, 3 h, and 12 h) when [$NiCl_2$] = 10 µM (left), 20 µM (middle), and 50 µM (right). S.D. n = 3.

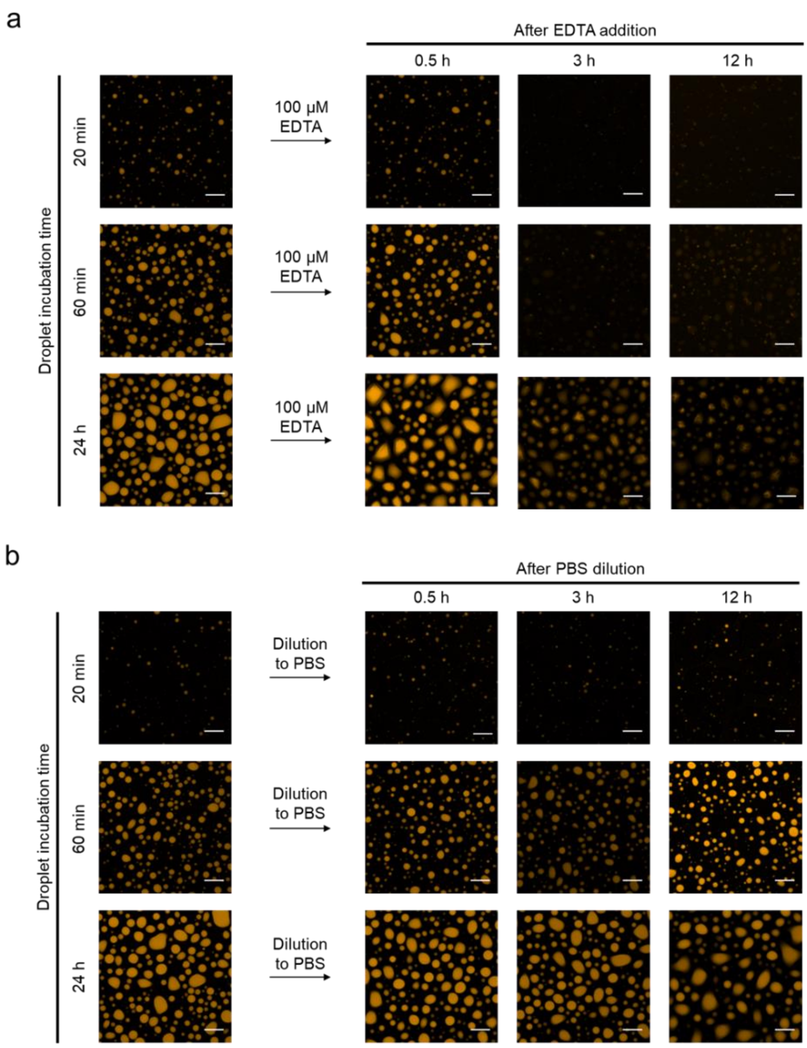


**Supplementary Figure 12** Reversibility of protein condensates with different incubation time. (a) EDTA treatment to differently incubated $Ni^{2+}$-PRM-SH3-6His condensates. (b) PBS dilution of differently incubated $Ni^{2+}$-PRM-SH3-6His condensates. Scale bars: 10 μm.

a

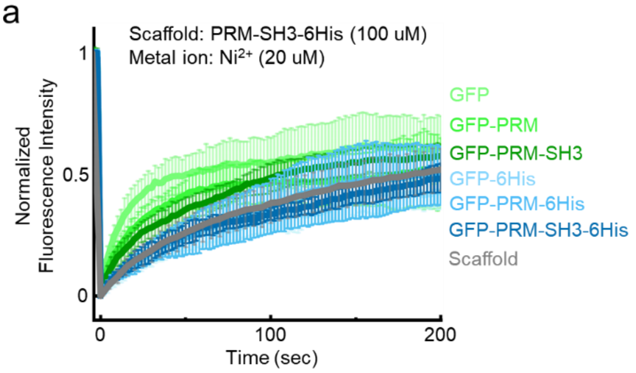


| Client protein | Mobile Fraction (%) | S.D. | $t_{1/2}$ (sec) | S.D. |
|---|---|---|---|---|
| GFP | 59.5 | 12.4 | 15 | 5.59 |
| GFP-PRM | 50.0 | 12.5 | 19 | 10.1 |
| GFP-PRM-SH3 | 60.5 | 5.76 | 40.6 | 10.4 |
| GFP-6His | 47.9 | 9.05 | 58.6 | 10.8 |
| GFP-PRM-6His | 60.2 | 6.90 | 78.3 | 26.1 |
| GFP-PRM-SH3-6His | 54.1 | 4.29 | 64.2 | 8.54 |
| Scaffold (PRM-SH3-6His) | 63.7 | 7.66 | 78.3 | 18.5 |

b

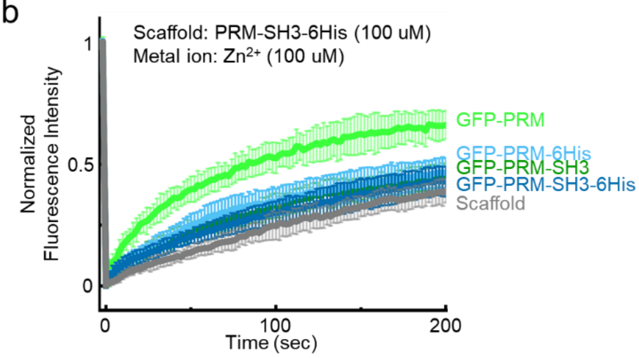


| Client protein | Mobile Fraction (%) | S.D. | $t_{1/2}$ (sec) | S.D. |
|---|---|---|---|---|
| GFP-PRM | 70.1 | 9.13 | 42.1 | 6.68 |
| GFP-PRM-SH3 | 52.4 | 5.91 | 68.5 | 15.8 |
| GFP-PRM-6His | 53.0 | 5.75 | 63.6 | 10.4 |
| GFP-PRM-SH3-6His | 52.2 | 7.97 | 77.4 | 11.1 |
| Scaffold (PRM-SH3-6His) | 59.8 | 9.04 | 148 | 19.5 |

c

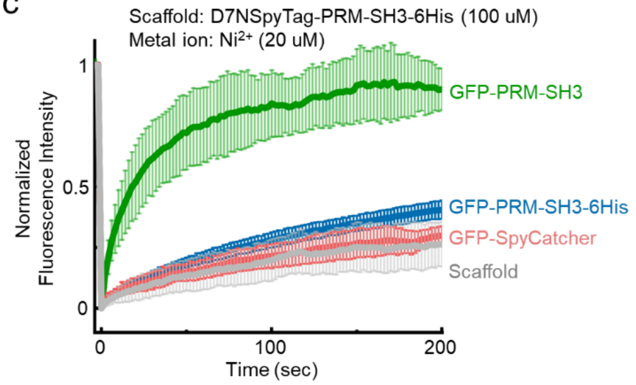


| Client protein | Mobile Fraction (%) | S.D. | $t_{1/2}$ (sec) | S.D. |
|---|---|---|---|---|
| GFP-PRM-SH3 | 90.3 | 11.1 | 20.9 | 8.23 |
| GFP-PRM-SH3-6His | 47.1 | 4.13 | 73.1 | 9.46 |
| GFP-SpyCatcher | 35.0 | 4.74 | 75.2 | 34.9 |
| Scaffold (D7NSpyTag-PRM-SH3-6His) | 34.2 | 14.5 | 80.0 | 39.8 |

**Supplementary Figure 13** Diffusivities of GFP-fused client protein variants inside condensates. (a) FRAP recovery profiles of GFP-fused clients inside PRM-SH3-6His condensates with $Ni^{2+}$. (b) FRAP recovery profiles of GFP-fused clients inside PRM-SH3-6His condensates with $Zn^{2+}$. (c) FRAP recovery profiles of GFP-fused clients inside D7NSpyTag-PRM-SH3-6His condensates with $Ni^{2+}$. S.D. from triplicate experiments with 12~31 condensates. Condensates were analyzed after 1 h at 25 ºC upon $Ni^{2+}$ addition.

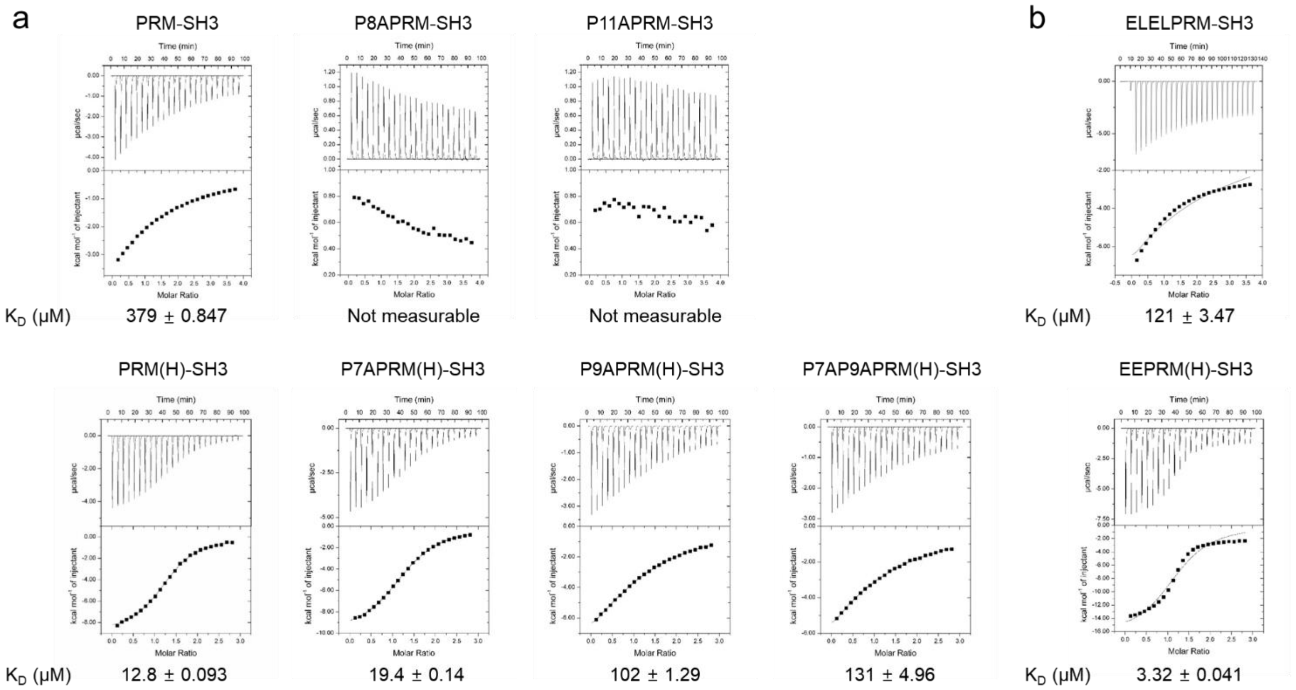


| Ligand | [Syringe] (μM) | [Cell] (μM) | $K_d$ (μM) | Affinity fold Increase |
|---|---|---|---|---|
| PRM | 3500 | 175 | 379 ± 0.847 | 1 |
| P8A | 4000 | 200 | N.A. | - |
| P11A | 4000 | 200 | N.A. | - |
| ELELPRM | 3000 | 150 | 121 ± 3.47 | 3.13 |
| PRM(H) | 1500 | 100 | 12.8 ± 0.093 | 29.6 |
| P7APRM(H) | 1500 | 100 | 19.4 ± 0.14 | 19.5 |
| P9APRM(H) | 1500 | 100 | 102 ± 1.29 | 3.70 |
| 2APRM(H) | 1500 | 100 | 131 ± 4.96 | 2.89 |
| EEPRM(H) | 1500 | 100 | 3.32 ± 0.041 | 114 |

**Supplementary Figure 14** ITC thermograms of various (a) PRM variants and (b) PRM(H) variants (syringe) addition to the SH3 domain (cell). PRM and PRM(H) variant peptides were chemically synthesized, while SH3 was recombinantly produced in *E. coli*. P8APRM/SH3 and P11APRM/SH3 thermograms were not suitable to fit for $K_D$ calculation due to their low binding affinities. Titration concentrations and relative binding affinities compared to the PRM/SH3 interaction are given in the below table.

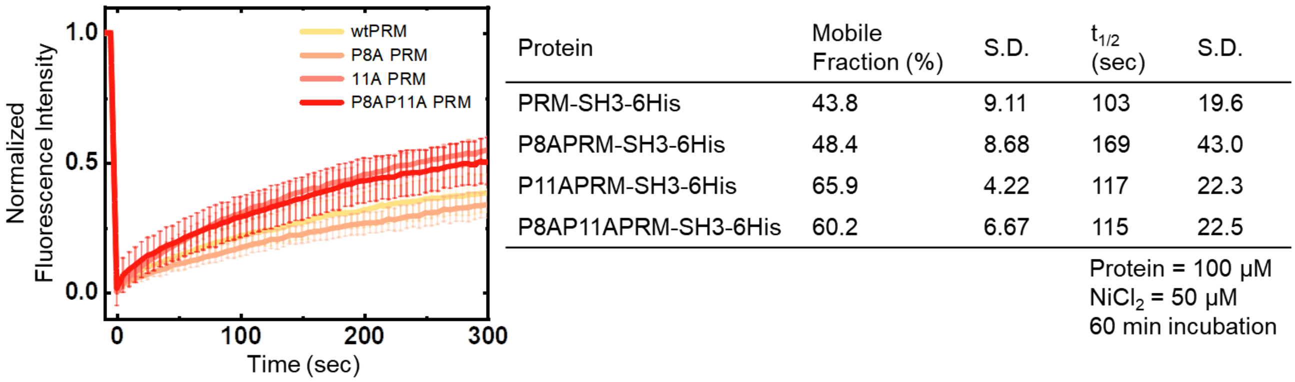


| Protein | Mobile Fraction (%) | S.D. | $t_{1/2}$ (sec) | S.D. |
|---|---|---|---|---|
| PRM-SH3-6His | 43.8 | 9.11 | 103 | 19.6 |
| P8APRM-SH3-6His | 48.4 | 8.68 | 169 | 43.0 |
| P11APRM-SH3-6His | 65.9 | 4.22 | 117 | 22.3 |
| P8AP11APRM-SH3-6His | 60.2 | 6.67 | 115 | 22.5 |

Protein = 100 μM
$NiCl_2$ = 50 μM
60 min incubation

**Supplementary Figure 15** FRAP recovery profiles of Pro-to-Ala mutated PRM-SH3-6His scaffold proteins inside condensates. S.D. from triplicate experiments with at least 17 condensates. Condensates were analyzed after 1 h at 25 ºC upon $Ni^{2+}$ addition.

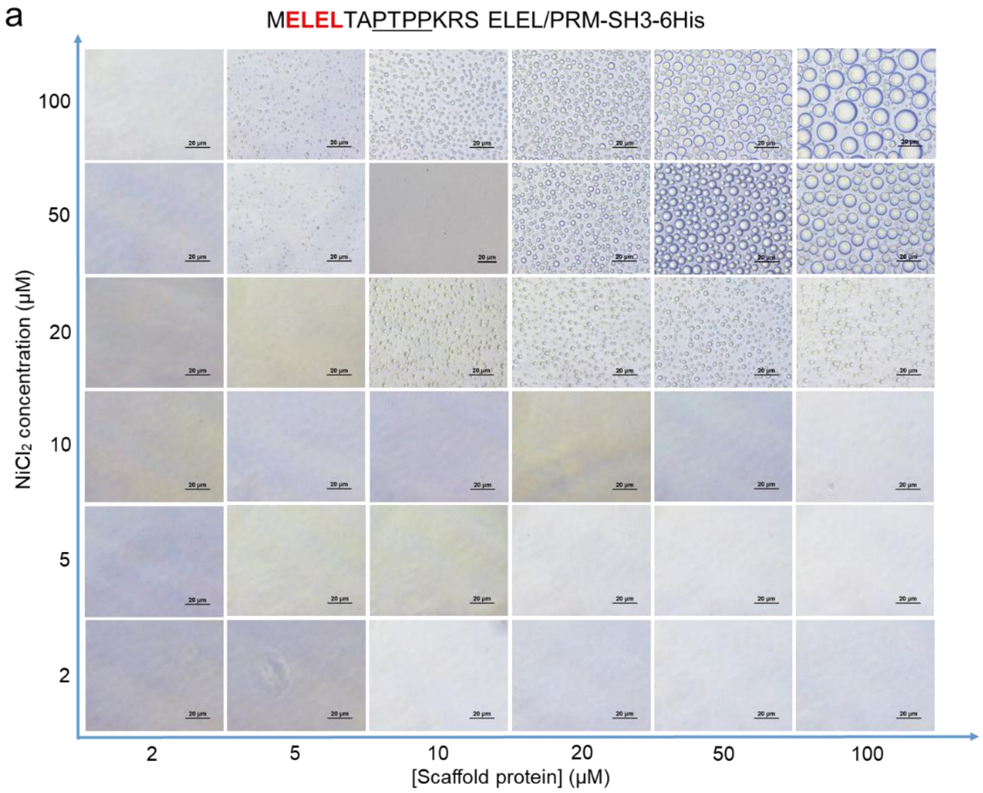


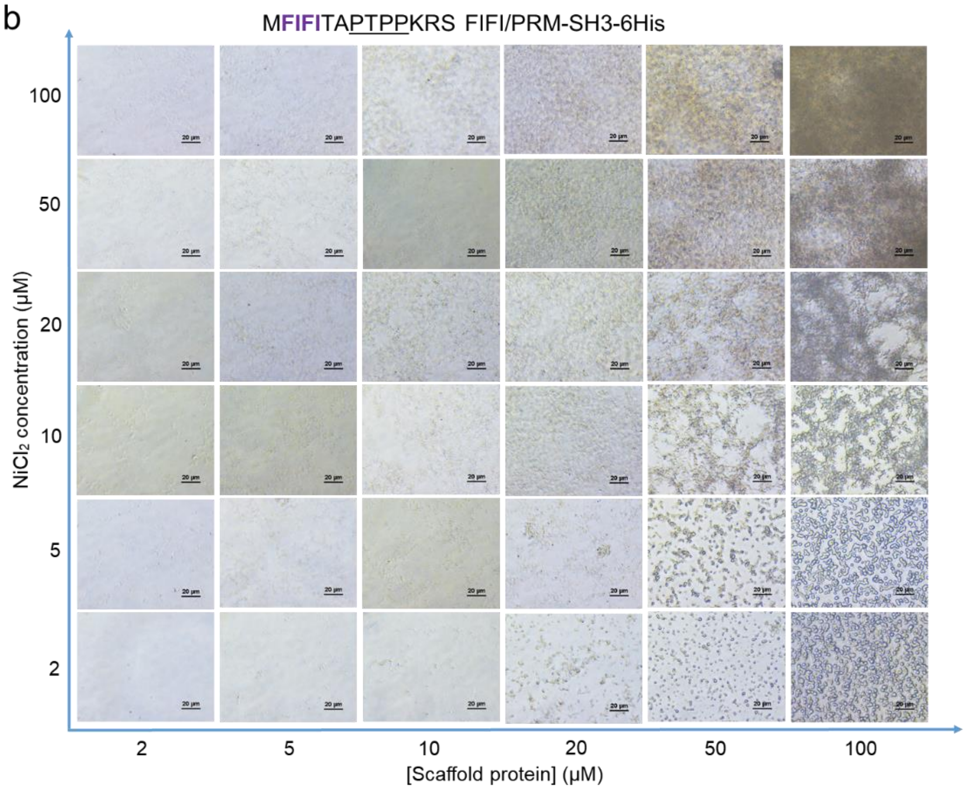


**Supplementary Figure 16** Optical images of phase separation diagrams of ligand mutated scaffold proteins. (continue)

c

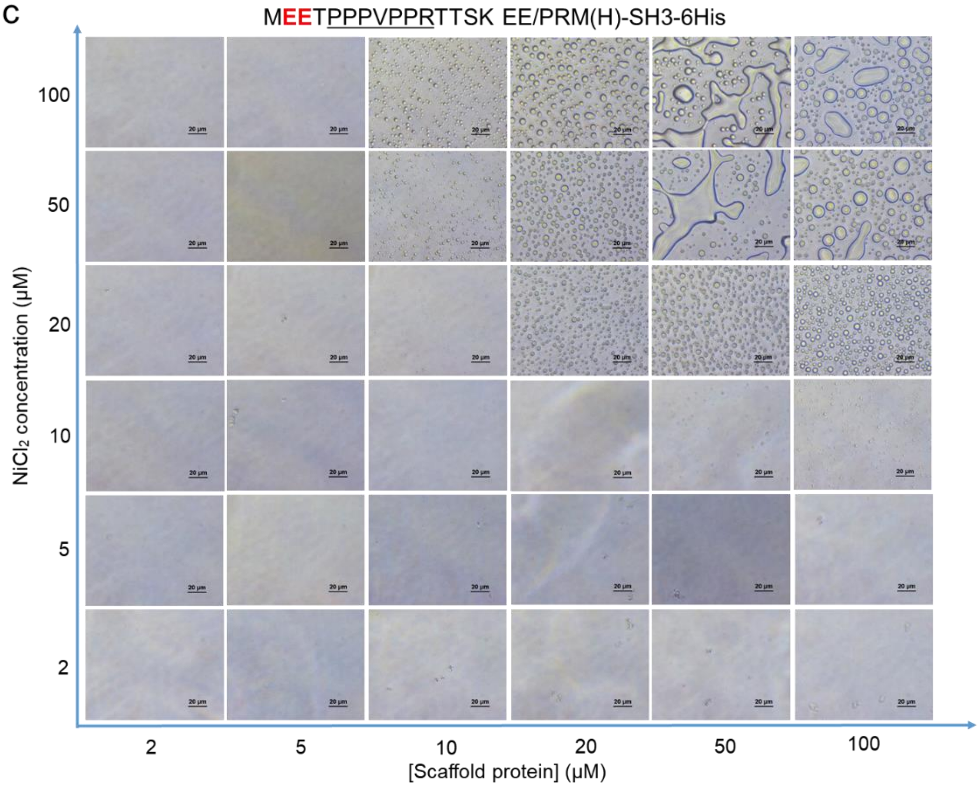


d

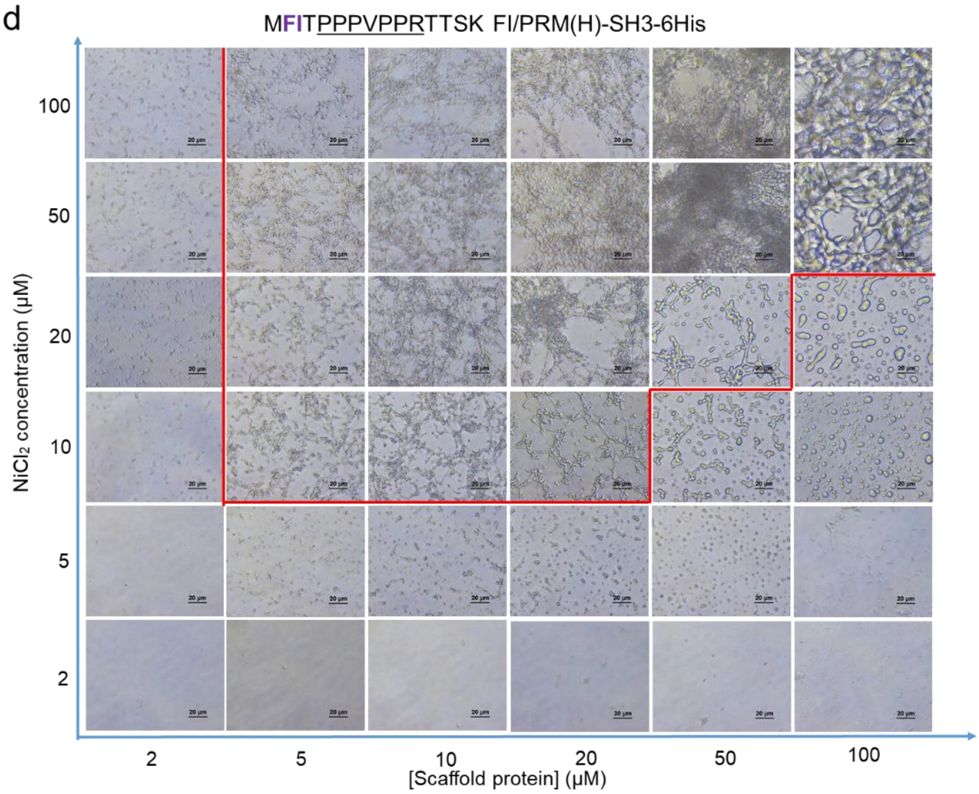


**Supplementary Figure 16** Optical images of phase separation diagrams of ligand mutated scaffold proteins. (continue)

e

MKKPSRPAPKPP KK/Ste20-NbpSH3-6His

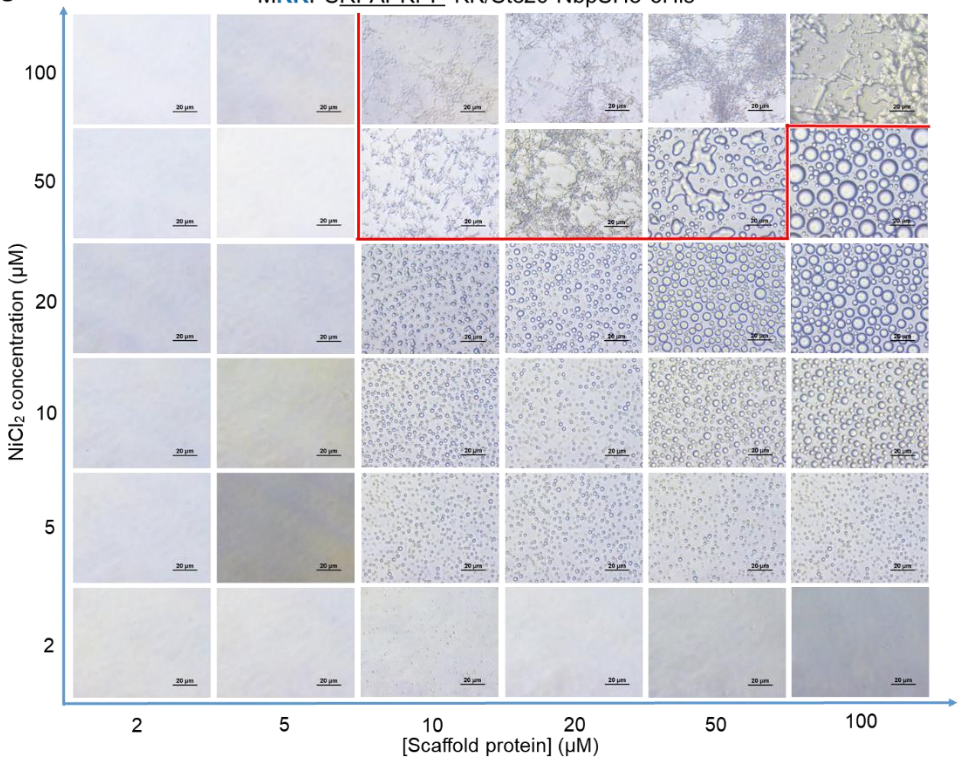


f

MEEPSRPAPKPP EE/Ste20-NbpSH3-6His

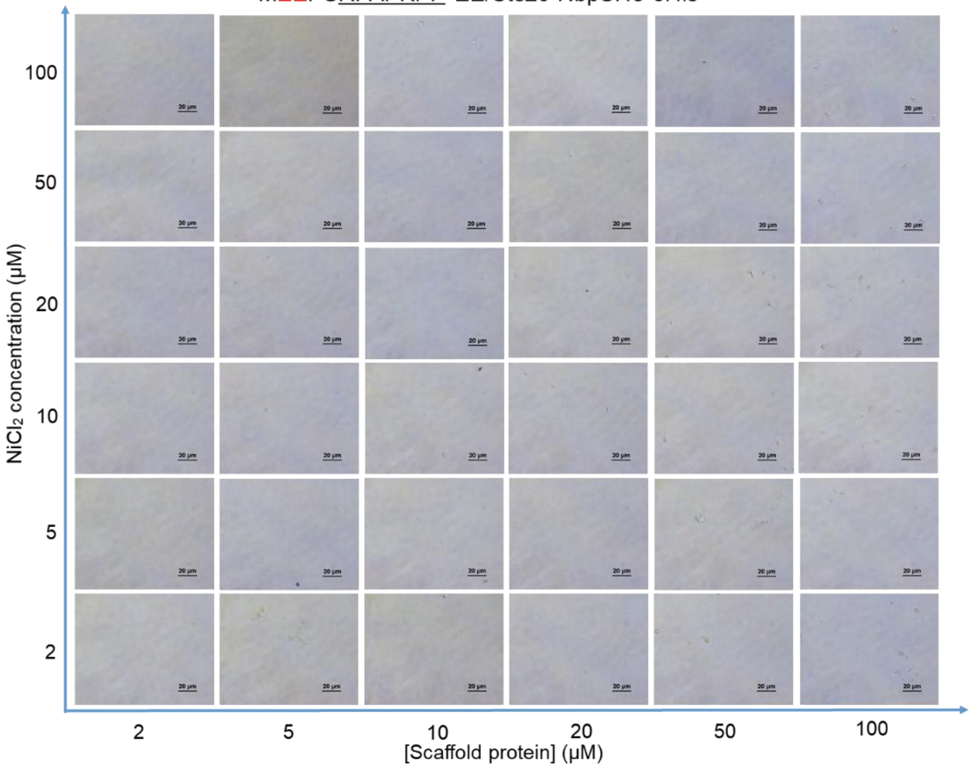


**Supplementary Figure 16** Optical images of phase separation diagrams of ligand mutated scaffold proteins. Mutated ligand sequences are shown above images. Phase diagram images of Fig. 4. Scale bars: 10 μm. Images were taken after 24 h upon $Ni^{2+}$ addition.

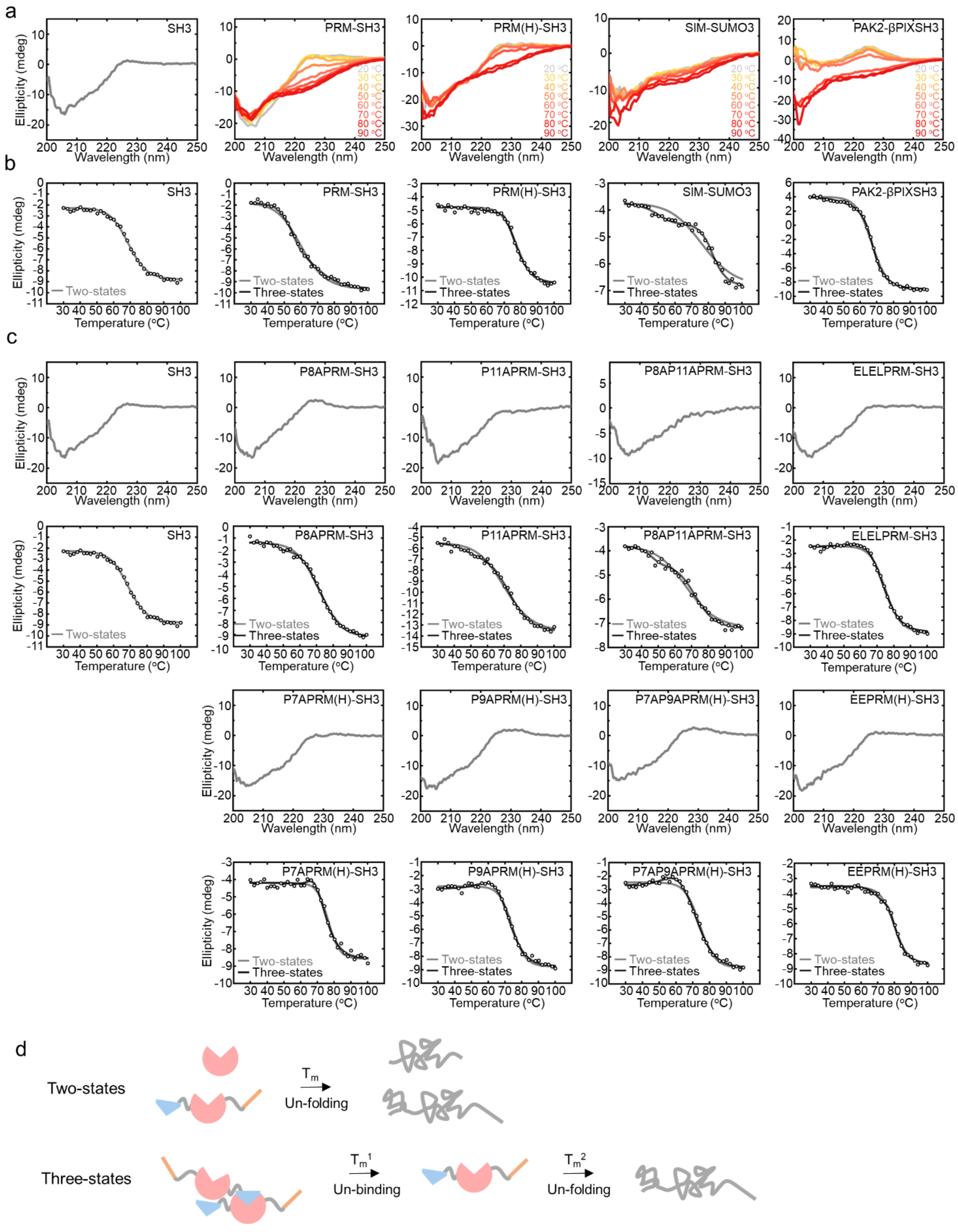


**Supplementary Figure 17** Temperature-variable circular dichroism (CD) analysis of various scaffold proteins. (continue)

| | Two-states | Three-states | |
|---|---|---|---|
| Protein | $T_m$ (°C) | $T_m^1$ (°C) | $T_m^2$ (°C) |
| SH3 | 68.83 ± 1.74 | | |
| PRM-SH3 | 60.09 ± 1.73 | 53.84 ± 0.57 | 66.68 ± 3.51 |
| P8APRM-SH3 | 71.32 ± 1.51 | 45.73 ± 3.18 | 72.76 ± 0.74 |
| P11APRM-SH3 | 70.65 ± 0.83 | 48.64 ± 3.22 | 73.55 ± 1.92 |
| P8AP11APRM-SH3 | 68.43 ± 0.34 | 46.89 ± 5.16 | 72.67 ± 1.68 |
| ELELPRM-SH3 | 74.96 ± 0.55 | 62.94 ± 3.41 | 71.16 ± 0.67 |
| PRM(H)-SH3 | 77.51 ± 1.11 | 66.54 ± 0.71 | 73.47 ± 0.78 |
| P7APRM(H)-SH3 | 76.55 ± 0.44 | 64.46 ± 1.09 | 73.68 ± 1.40 |
| P9APRM(H)-SH3 | 74.36 ± 1.06 | 59.84 ± 0.34 | 71.50 ± 0.99 |
| P7AP9APRM(H)-SH3 | 74.59 ± 0.97 | 59.11 ± 1.20 | 72.47 ± 0.67 |
| EEPRM(H)-SH3 | 79.96 ± 0.70 | 73.81 ± 1.59 | 77.47 ± 0.62 |
| SIM-SUMO3 | 75.43 ± 1.69 | 48.63 ± 5.28 | 82.89 ± 1.86 |
| PAK2-βPIXSH3 | 67.03 ± 0.88 | 65.89 ± 0.34 | 67.50 ± 1.16 |

**Supplementary Figure 17** Temperature-variable circular dichroism (CD) analysis of various scaffold proteins. (a) Ellipticity profiles (from 200 nm to 250 nm) and (b) ellipticity changes at 222 nm as a function of temperature of LLPS scaffold proteins and free SH3. (c) Ellipticity profiles and changes at 222 nm for Pro-to-Ala and acidic mutation scaffolds. Two-fitting lines (two-state in gray and three-state in black) are indicated. (d) Schematic diagram of two-state and three-state transition models. Calculated unfolding $T_m$ in a two-state transition (folding/unfolding) model and scaffold unbinding $T_m^1$ and unfolding $T_m^2$ in a three-state transition (protein unbinding and unfolding) model are given in the below table. All proteins were analyzed at 20 μM concentration in 50 mM sodium phosphate pH 7.4. S.D. n = 3.

**Note:** Ellipticity profiles were mostly unchanged by tested mutations, indicating structural stability of various binding peptide mutants. Mutations to the binding peptides (PRM and PRM(H)) rather than folded globular domain (SH3) might have only a minimal effects on protein folding/stability. On the other hand, $T_m$ (particularly $T_m^1$) values were widely varied by mutations. For example, $T_m^1$ values clearly decreased by (binding weakening) P-to-A mutations, while increased by (binding strengthening) K-to-E mutations (Supplementary Figs. 14 and 17). Raw data and thermodynamic parameters are given in Source Data.

**Data analysis:** The data fitting was conducted by following the procedures from a previous report[1] with slight modification for a three-state model. The midpoint transition temperature ($T_m$) of un-

folding was calculated by fitting measured ellipticities at 222 nm at a given temperature to the calculated ellipticity. A two-state transition model was used for a single $T_m$ calculation.

$$\mathrm{F} \overset{K}{\leftrightarrow} \mathrm{U},\ K = [U]/[F] = e^{-\Delta G/RT}, \tag{1}$$

where [F] and [U] are the concentrations of folded and unfolded proteins, respectively.

$$\alpha = [F]/([F]+[U]) = 1/(1+K) = (\theta_t - \theta_U)/(\theta_F - \theta_U), \tag{2}$$

where α is the fraction of folded proteins, $\theta_t$ is a measured ellipticity at any temperature, $\theta_F$ is the ellipticity where 100 % of proteins exist in a folded form, and $\theta_U$ is the ellipticity where 100 % of proteins exist in an unfolded form.

Since $\Delta C_p$ is difficult to estimate from CD measurements, we set $\Delta C_p$ = 0 in this analysis.

$$\Delta \mathrm{G} = \Delta \mathrm{H}(1 - T/T_m) - \Delta C_p((T_m - T) + T ln(T/T_m)) \approx \Delta \mathrm{H}(1 - T/T_m), \tag{3}$$

where the midpoint transition temperature of unfolding $T_m$ is the temperature at $K = 1$.

A three-state transition model was used to consider both un-binding $T_m^1$ (e.g. interactions between PRM and SH3) and un-folding $T_m^2$ (e.g. SH3) processes (Supplementary Fig 17d).

$\mathrm{D} \overset{K_1}{\leftrightarrow} 2\mathrm{M} \overset{K_2}{\leftrightarrow} 2\mathrm{U}$ (D: dimer, M: monomer, U: un-folded monomer); Three-state equilibrium was simplified with two independent equilibrium states in our model.

$$\mathrm{D} \overset{K_1}{\leftrightarrow} 2\mathrm{M}, \text{ and } \mathrm{M} \overset{K_2}{\leftrightarrow} \mathrm{U}$$

For the simple modeling, we assumed that two equilibriums are independently governed by given equilibrium constants, and each equilibrium shift also contributes to the change of ellipticity with fraction $\gamma_1$ and $\gamma_2$ $(\gamma_1 + \gamma_2 = 1)$.

$$K_1 = [M]^2/[D] = [M]([M]/[D]) = [M]\left(e^{-\Delta G_1/RT}\right), \text{and } K_2 = [U]/[M] = e^{-\Delta G_2/RT} \tag{4}$$

where [M], [D], and [U] are the concentrations of monomer, dimer, and un-folded monomer proteins (when fitting $K_1$, we set [M] ≈ the initial total protein concentration 20 μM as a constraint for ease of fitting).

$$\alpha_1 = 2[D]/(2[D]+[M]) \text{ and } \alpha_2 = [M]/([M]+[U])$$

$$(\alpha_1\gamma_1 + \alpha_2\gamma_2) = (\theta_t - \theta_U)/(\theta_D - \theta_U), \tag{5}$$

where $\alpha_1$ is the fraction of monomeric proteins (or domains) which exists in dimeric forms in the D $\overset{K_1}{\leftrightarrow}$ 2M two-state system, $\alpha_2$ is the fraction of folded monomeric proteins in the M $\overset{K_2}{\leftrightarrow}$ U two-state system, $\theta_t$ is a measured ellipticity at any temperature, $\theta_D$ is the ellipticity where 100 % of proteins exist in a dimeric form, and $\theta_U$ is the ellipticity where 100 % of proteins exist in monomeric and unfolded forms.

Again, we set all $\Delta C_p$ = 0 in this analysis.

$$\Delta G_1 = \Delta H_1(1 - T/T_{m^1}) - \Delta C_{p^1}((T_{m^1} - T) + Tln(T/T_{m^1})) \approx \Delta H_1(1 - T/T_{m^1}), \quad (6)$$

$$\Delta G_2 = \Delta H_2(1 - T/T_{m^2}) - \Delta C_{p^2}((T_{m^2} - T) + Tln(T/T_{m^2})) \approx \Delta H_2(1 - T/T_{m^2}), \quad (7)$$

where the midpoint transition temperature of un-binding (dissociation) $T_{m^1}$ is the temperature at $K_1$ = 1, and the midpoint transition temperature of monomer un-folding $T_{m^2}$ is the temperature at $K_2$ = 1. Estimated ellipticity calculations and curve fittings were carried out by using a Excel® (Microsoft) nonlinear least squares analysis tool with given initial parameters (ΔH, $\theta_F$, $\theta_D$ and $\theta_U$) based on CD and ITC experiment results.

The Fitting data are provided in Source Data.

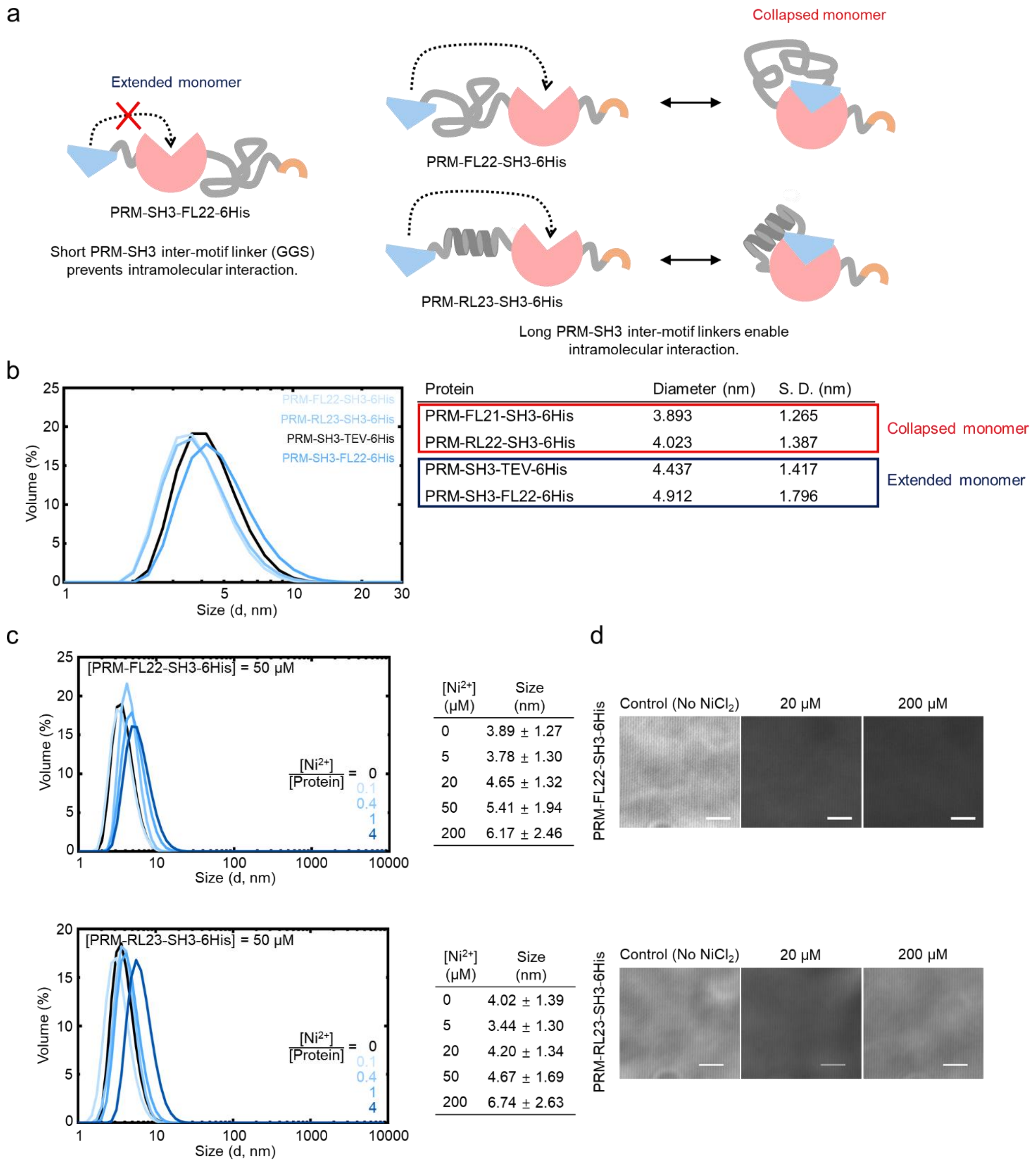


**Supplementary Figure 18** Inter-motif linkers between PRM and SH3 (a) Schematic representations of PRM-SH3-Linker-6His with an extended monomer structure (left) and PRM-Linker-SH3-6His with a collapsed monomer structure. (b) DLS size distribution profiles of PRM-SH3 linker variants. Average sizes of are indicated in the right table. s.d. from n = 3. (c) DLS size distribution profiles of PRM-FL22-SH3 and PRM-RL23-SH3 with varying ratios of $Ni^{2+}$. (d) Optical microscopy images of 50 µM PRM-FL22-SH3 (upper) and PRM-RL23-SH3 (lower) with 20 µM or 200 µM $NiCl_2$. Images were taken after 12 h incubation at 25 °C upon LLPS. Scale bars: 10 µm.

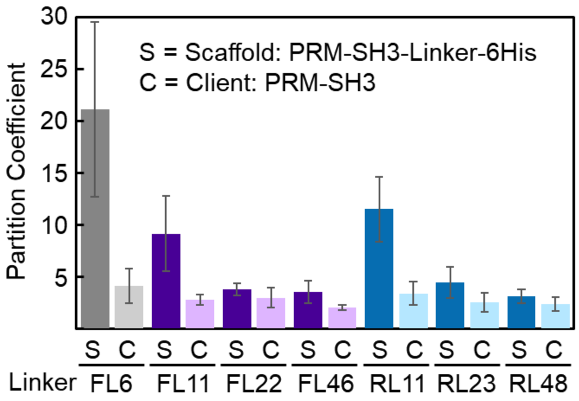


**Supplementary Figure 19** Scaffold and client PC values for various linker added scaffold proteins. 100 µM of scaffold proteins were mixed with 50 µM $NiCl_2$, and condensates were imaged after 30 min upon $NiCl_2$ addition. Error bars: 1 s.d. from triplicate experiments with at least 195 condensates.

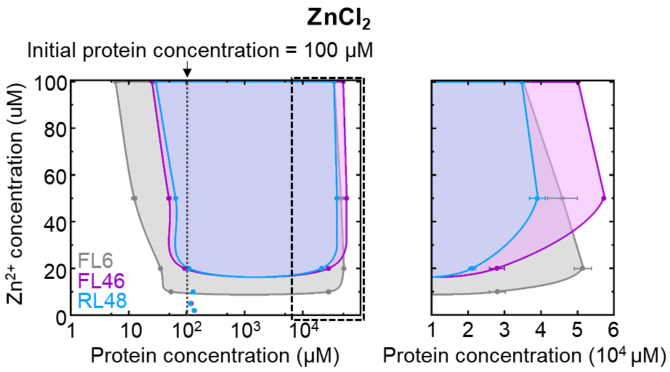


| [Zn$^{2+}$] (μM) | PRM-SH3-FL6 | | PRM-SH3-FL46 | | PRM-SH3-RL48 | |
|---|---|---|---|---|---|---|
| | [Dilute] (μM) | [Dense] (μM) | [Dilute] (μM) | [Dense] (μM) | [Dilute] (μM) | [Dense] (μM) |
| 2 | - | - | - | - | - | - |
| 5 | - | - | - | - | - | - |
| 10 | 53.6 ± 2.07 | 28000 ± 2160 | - | - | - | - |
| 20 | 35.3 ± 1.30 | 51400 ± 2360 | 91.8 ± 4.72 | 27900 ± 2170 | 109 ± 1.81 | 21200 ± 932 |
| 50 | 12.4 ± 1.24 | 45900 ± 4090 | 49.3 ± 3.73 | 57200 ± 207 | 64.4 ± 1.17 | 39000 ± 2120 |
| 100 | 5.88 ± 0.860 | 35100 ± 2000 | 25.1 ± 1.29 | 50200 ± 318 | 29.3 ± 0.0978 | 34700 ± 939 |

**Supplementary Figure 20** The phase diagrams of PRM-SH3-Linker-6His with dense and dilute phase protein concentrations as a function of Zn$^{2+}$. Left arms exhibit dilute phase concentrations and right arms for dense phase concentrations of the scaffold (PRM-SH3-6His) protein. The magnified version of the right arms (dash box in the left diagram) is drawn (right diagram). Concentrations are summarized in the below table. S.D. from at least 2 independent experiments. The FL6 data are same as Supplementary Fig. 10, but included for better comparison with the FL46 and RL48 data.

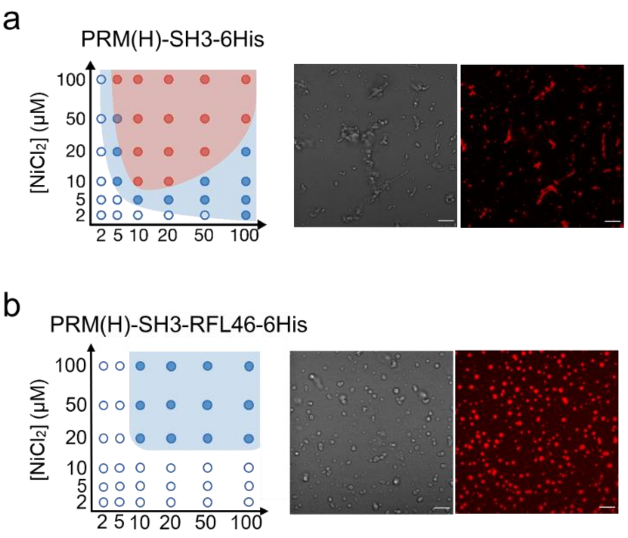


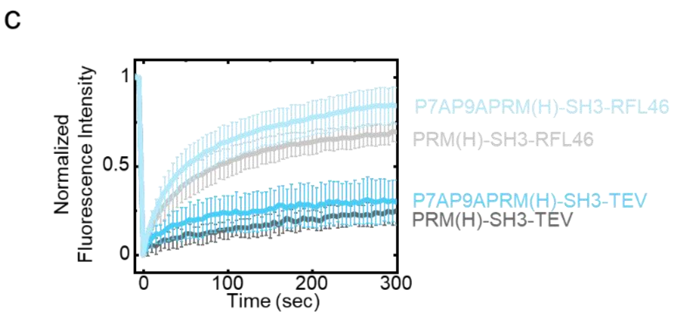


| Protein | Mobile Fraction (%) | S.D. | $t_{1/2}$ (sec) | S.D. |
|---|---|---|---|---|
| PRM(H)-SH3-6His | 26.9 | 5.22 | 99.9 | 40.4 |
| PRM(H)-SH3-RFL46-6His | 69.9 | 3.93 | 48.8 | 14.5 |
| P7AP9APRM(H)-SH3-6His | 32.0 | 10.5 | 64.7 | 52.6 |
| P7AP9APRM(H)-SH3-RFL46-6His | 82.7 | 9.01 | 43.2 | 10.0 |

Protein = 100 μM
$NiCl_2$ = 20 μM
60 min incubation

**Supplementary Figure 21** Condensate morphology shift by scaffold linker addition. (a) A phase diagram and optical images of PRM(H)-SH3-6His condensates (protein 100 μM and $Ni^{2+}$ 20 μM). DIC and optical images were taken after 24 h upon condensate formation. (b) A phase diagram and optical images of PRM(H)-SH3-RFL46-6His condensates (protein 100 μM and $Ni^{2+}$ 20 μM). Scale bars: 10 μm. (c) FRAP recovery profiles of PRM(H)-SH3-6His linker and Pro-to-Ala scaffold variants. S.D. from triplicate experiments with at least 10 condensates.

**Note:** Pro-to-Ala mutated P7AP9APRM(H)-SH3-6His (Fig. 4b) condensates also showed increased diffusivity by inserting the long RFL46 peptide linker.

RFL linker: GSKESGSVSSEQLAQFRSLDEFEGKSSGSGSESKSTETSGGSGSLE

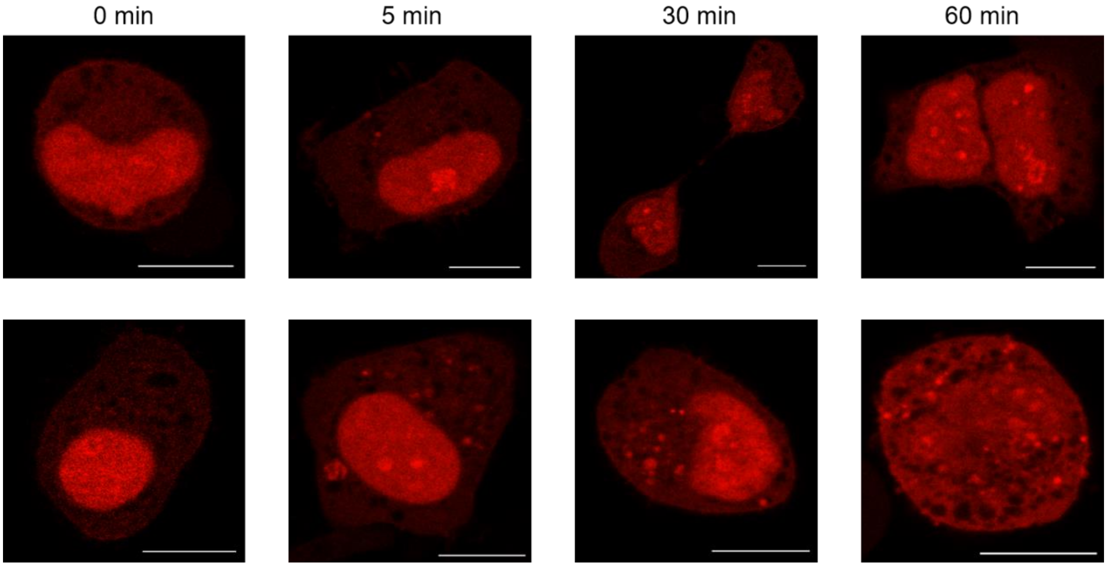


**Supplementary Figure 22** Time-dependent increase of the $Zn^{2+}$-mediated formation of mCh-PRM-SH3-6His condensates in HeLa cells. Scale bars: 10 μm.

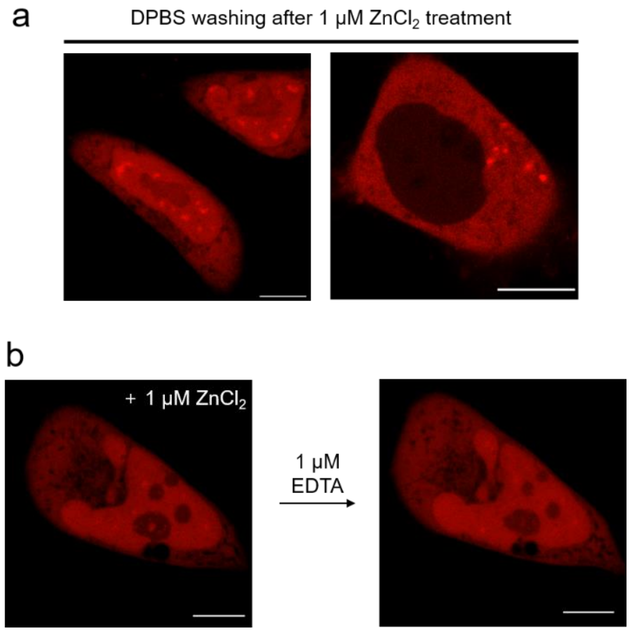


**Supplementary Figure 23** Irreversible formation of $Zn^{2+}$-mediated mCh-PRM-SH3-6His puncta. (a) Fluorescence images of mCh-PRM-SH3-6His condensates in HeLa cells with DPBS washing after 1 μM of $ZnCl_2$ treatment. (b) Fluorescence images of mCh-PRM-SH3-6His condensates in HeLa cells with EDTA treatment after 1 μM of $ZnCl_2$ treatment. Scale bars: 10 μm.

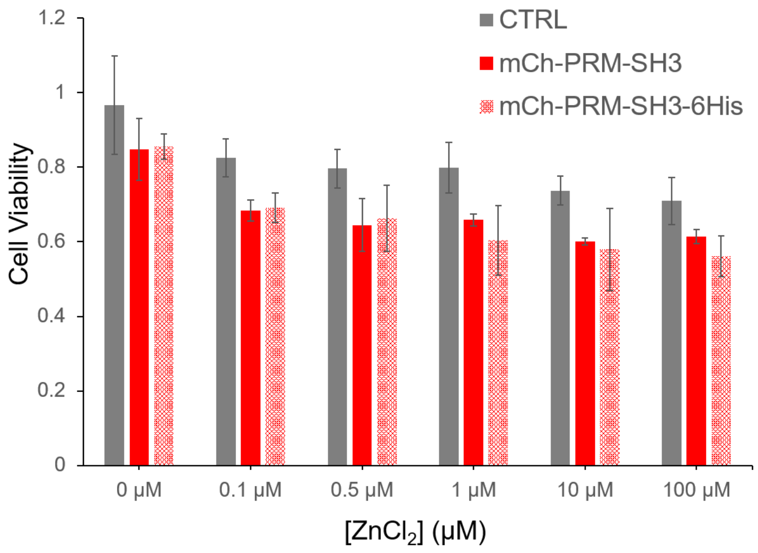


**Supplementary Figure 24** Effects of mCherry-PRM-SH3 protein and puncta formation in HeLa cells as a function of $ZnCl_2$ concentration measured by MTT assay. S. D. n = 3

## Supplementary Tables

**Supplementary Table 1** Partition coefficients of four scaffold proteins. (Data for Fig. 1f)

| Client | P.C. | S.D. |
|---|---|---|
| PRM-SH3 | 12.1 | 2.31 |
| PRM(H)-SH3-TEV | 31.8 | 13.1 |
| SIM-SUMO3 | 4.19 | 0.46 |
| PAK2-βPIXSH3 | 12.8 | 3.46 |

[Protein] = 100 μM, [$Ni^{2+}$] = 20 μM (50 μM for SIM-SUMO3), 60 min incubation
Standard deviation (S.D.) from three independent experiments.

**Supplementary Table 2** Mobile fraction and $t_{1/2}$ values of FRAP analysis for four scaffold proteins (Data for Fig. 1g)

| Protein | Mobile Fraction (%) | S.D. | $t_{1/2}$ (sec) | S.D. |
|---|---|---|---|---|
| PRM-SH3 | 63.7 | 7.66 | 78.3 | 18.5 |
| PRM(H)-SH3-TEV | 26.9 | 5.22 | 99.9 | 40.4 |
| SIM-SUMO3 | 35.4 | 7.25 | 77.9 | 22.4 |
| PAK2-βPIXSH3 | 7.74 | 3.02 | 58.3 | 53.2 |

[Protein] = 100 μM, [$Ni^{2+}$] = 20 μM (50 μM for SIM-SUMO3), 60 min incubation
S.D. from three independent experiments.

**Supplementary Table 3** Mobile fraction and $t_{1/2}$ values of FRAP analysis for four metal ions (Data for Fig. 2b)

| Metal ion | Mobile Fraction (%) | S.D | $t_{1/2}$ (sec) | S.D. |
|---|---|---|---|---|
| $Ni^{2+}$ | 36.7 | 21.9 | 193 | 119 |
| $Zn^{2+}$ | 75.8 | 8.93 | 96.4 | 66.7 |
| $Co^{2+}$ | 68.6 | 10.0 | 137 | 48.3 |
| $Cu^{2+}$ | 85.8 | 6.08 | 45.9 | 14.5 |

[Protein] = 100 μM, [Metal ion] = 100 μM, 60 min incubation
S.D. from three independent experiments.

**Supplementary Table 4** Mobile fraction and $t_{1/2}$ values of FRAP analysis for linker added PRM-SH3-Linker-6His scaffold proteins (Data for Fig. 5c)

| Inter-motif linker | Mobile Fraction (%) | S.D | $t_{1/2}$ (sec) | S.D. |
|---|---|---|---|---|
| FL6 | 26.9 | 6.56 | 115 | 61.3 |
| FL11 | 50.8 | 7.00 | 109 | 23.0 |
| FL22 | 70.8 | 4.33 | 37.7 | 6.72 |
| FL46 | 81.3 | 8.93 | 34.8 | 4.04 |
| RL11 | 47.6 | 5.88 | 91.7 | 19.5 |
| RL23 | 86.0 | 3.44 | 29.4 | 6.27 |
| RL48 | 73.4 | 5.73 | 28.9 | 5.85 |

[Protein] = 100 μM, [$Ni^{2+}$] = 50 μM, 30 min incubation
S.D. from three independent experiments.

**Supplementary Table 5** Mobile fraction and $t_{1/2}$ values of FRAP analysis for the PRM-SH3 client inside linker added PRM-SH3-Linker-6His condensates (Data for Fig. 5c)

| Inter-motif linker | Mobile Fraction (%) | S.D | $t_{1/2}$ (sec) | S.D. |
|---|---|---|---|---|
| FL6 | 61.7 | 6.08 | 63.8 | 11.8 |
| FL11 | 74.6 | 5.68 | 28.3 | 3.57 |
| FL22 | 85.7 | 4.35 | 22.1 | 4.75 |
| FL46 | 82.1 | 3.64 | 11.2 | 2.71 |
| RL11 | 74.8 | 8.17 | 26.1 | 3.41 |
| RL23 | 86.9 | 6.05 | 12.7 | 5.00 |
| RL48 | 85.2 | 5.25 | 10.8 | 3.43 |

[Protein] = 100 μM, [$Ni^{2+}$] = 50 μM, 30 min incubation
S.D. from three independent experiments.

**Supplementary Table 6** Mobile fraction and $t_{1/2}$ values of FRAP analysis for cellular condensates (Data for Fig. 6)

| Protein | Mobile Fraction (%) | S.D. | $t_{1/2}$ (sec) | S.D. |
|---|---|---|---|---|
| mCh-PRM-SH3-6His | 92.8 | 21.1 | 53.8 | 32.6 |
| mCh-PRM(H)-SH3-6His | 41.5 | 21.9 | 31.9 | 25.4 |

[$Zn^{2+}$] = 10 μM, 60 min incubation
S.D. from 6 – 8 cellular droplets.

**Protein Sequences**

Blue: peptide ligand, Red: receptor, Purple: receptor-6His inter-motif linker (TEV protease site underlined), Green: additional motifs or proteins

| Protein/Ligand | Amino acid sequence | Note |
|---|---|---|
| **(PRM variant)-SH3-6His** | (PRMvariant)GGSDLNMPAYVKFNYMAEREDELSLIKGTKVIVMEKSSDGWWRGSYNGQVGWFPSNYVTEEGDSPLGSENLYFQGLEHHHHHH | PRM: Human Abl1 residues 606-618<br>SH3: Human Nck1 second SH3 domain residues 106-168, C169S mutation to prevent disulfide bond formation[2] |
| **PRM** | MKKKKTAPTPPKRS | PRM (wild-type) |
| **P8APRM** | MKKKKTAATPPKRS | PRM variant |
| **P11APRM** | MKKKKTAPTPAKRS | PRM variant |
| **P8AP11APRM** | MKKKKTAATPAKRS | PRM variant |
| **ELEL/PRM** | MELELTAPTPPKRS | PRM variant |
| **FIFI/PRM** | MFIFITAPTPPKRS | PRM variant |
| **PRM-(Linker)-SH3-6His** | MKKKKTAPTPPKRS(Linker)DLNMPAYVKFNYMAEREDELSLIKGTKVIVMEKSSDGWWRGSYNGQVGWFPSNYVTEEGDSPLLEHHHHHH | |
| **PRM-SH3-(Linker)-6His** | MKKKKTAPTPPKRSGGSDLNMPAYVKFNYMAEREDELSLIKGTKVIVMEKSSDGWWRGSYNGQVGWFPSNYVTEEGDSPL(Linker)HHHHHH | |
| **FL6** | GSGSLE | Linker variant |
| **FL11** | GSGGSGSGSLE | Linker variant |
| **FL21** | GSGGSGGSGSGSGGSGGSGSL | Linker variant |
| **FL22** | GSGGSGGSGSGSGGSGGSGSLE | Linker variant |
| **FL46** | GSGGSGGSGSGSGGSGGSGGSGSGGSGGSGGSGSGSGGSGGSGSLE | Linker variant |
| **RL11** | GSEAAAKGSLE | Linker variant |
| **RL22** | GSEAAAKEAAAKEAAAKGSGSL | Linker variant |
| **RL23** | GSEAAAKEAAAKEAAAKGSGSLE | Linker variant |
| **RL48** | GSEAAAKEAAAKEAAAKEAAAKEAAAKEAAAKEAAAKEAAAKGSGSLE | Linker variant |
| **RFL45** | GSKESGSVSSEQLAQFRSLDEFEGKSSGSGSESKSTETSGGSGSL | Linker variant |
| **RFL46** | GSKESGSVSSEQLAQFRSLDEFEGKSSGSGSESKSTETSGGSGSLE | Linker variant |
| **(PRM(H) variant)-SH3-6His** | (PRM(H)variant)GGSDLNMPAYVKFNYMAEREDELSLIKGTKVIVMEKSSDGWWRGSYNGQVGWFPSNYVTEEGDSPLGSENLYFQGLEHHHHHH | PRM(H): Human Disks large-associated protein 2 residues 610-623[2] |
| **PRM(H)** | MKKTPPPVPPRTTSK | PRM(H) (wild-type) |
| **P7APRM(H)** | MKKTPPAVPPRTTSK | PRM(H) variant |
| **P9APRM(H)** | MKKTPPPVAPRTTSK | PRM(H) variant |
| **P7AP9APRM(H)** | MKKTPPAVAPRTTSK | PRM(H) variant |
| **EE/PRM(H)** | MEETPPPVPPRTTSK | PRM(H) variant |
| **FI/PRM(H)** | MFITPPPVPPRTTSK | PRM(H) variant |

| | | |
|---|---|---|
| **(PRM(H) variant)-SH3-RFL46-6His** | (PRM(H)variant)GGSDLNMPAYVKFNYMAEREDELSLIKGTKVIVMEKSSDGWWRGSYNGQVGWFPSNYVTEEGDSPLGSKESGSVSSEQLAQFRSLDEFEGKSSGSGSESKSTETSGGSGSLEHHHHHH | For Supplementary Fig. 14 |
| **SIM-SUMO-6His** | MKVDVIDLTIESSSDEEEDPPAKRGSMSEEKPKEGVKTENDHINLKVAGQDGSVVQFKIKRHTPLSKLMKAYCERQGLSMRQIRFRFDGQPINETDTPAQLEMEDEDTIDVFQQQTVVGSENLYFQGLEHHHHHH | SIM: Human E3 SUMO-protein ligase PIAS2 residues 466-488<br>SUMO: Human Small ubiquitin-related modifier 3 residues 1-92, G91V and G92V mutations to prevent processing by proteases[3] |
| **PAK2-βPIXSH3-6His** | MEETAPPVIAPRPDHTKSIYTRSVIGGSGPLGSVVRAKFNFQQTNEDELSFSKGDVIHVTRVEEGGWWEGTHNGRTGWFPSNYVREIGSENLYFQGLEHHHHHH | PAK2: Human Serine/threonine-protein kinase PAK 2 residues 176-199<br>βPIXSH3: Rat Rho guanine nucleotide exchange factor 7 residues 10-63[4] |
| **(Ste20 variant)-NbpSH3-6His** | (Ste20variant)GGSIVNQRAVALYDFEPENDNELRLAEGDIVFISYKHGQGWLVAENESGSKTGLVPEEFVSYIQPEGSENLYFQGLEHHHHHH | Ste20: Yeast Serine/threonine-protein kinase STE20 residues 470~480<br>NbpSH3: Yeast NAP1-binding protein residues 110-172[5] |
| **Ste20** | MFIPSRPAPKPP | Ste20 (wild-type) |
| **KK/Ste20** | MKKPSRPAPKPP | Ste20 variant |
| **EE/Ste20** | MEEPSRPAPKPP | Ste20 variant |
| **D7NSpyTag-PRM-SH3-6His** | MAHIVMVNAYKPTKGGSKKKKTAPTPPKRSGGSDLNMPAYVKFNYMAEREDELSLIKGTKVIVMEKSSDGWWRGSYNGQVGWFPSNYVTEEGDSPLGSENLYFQGLEHHHHHH | SpyTag D7N mutant |
| **GFP-6His** | MKGEELFTGVVPILVELDGDVNGHEFSVRGEGEGDATIGKLTLKFICTTGKLPVPWPTLVTTLTYGVQCFSRYPDHMKRHDFFKSAMPEGYVQERTISFKDDGKYKTRAVVKFEGDTLVNRIELKGTDFKEDGNILGHKLEYNFNSHDVYITADKQENGIKAEFTVRHNVEDGSVQLADHYQQNTPIGDGPVLLPDNHYLSTQTVLSKDPNEKRDHMVLHEYVNAAGITGSENLYFQGLEHHHHHH | GFP: The (-9) charge variant of superfolder GFP[6] |
| **GFP-PRM-6His** | MKKKKTAPTPPKRSGGSKGEELFTGVVPILVELDGDVNGHEFSVRGEGEGDATIGKLTLKFICTTGKLPVPWPTLVTTLTYGVQCFSRYPDHMKRHDFFKSAMPEGYVQERTISFKDDGKYKTRAVVKFEGDTLVNRIELKGTDFKEDGNILGHKLEYNFNSHDVYITADKQENGIKAEFTVRHNVEDGSVQLADHYQQNTPIGDGPVLLPDNHYLSTQTVLSKDPNEKRDHMVLHEYVNAAGITGSENLYFQGLEHHHHHH | |

| | | |
|---|---|---|
| **GFP-PRM-SH3-6His** | MKGEELFTGVVPILVELDGDVNGHEFS<br>VRGEGEGDATIGKLTLKFICTTGKLPVP<br>WPTLVTTLTYGVQCFSRYPDHMKRHDF<br>FKSAMPEGYVQERTISFKDDGKYKTRA<br>VVKFEGDTLVNRIELKGTDFKEDGNILG<br>HKLEYNFNSHDVYITADKQENGIKAEFT<br>VRHNVEDGSVQLADHYQQNTPIGDGPV<br>LLPDNHYLSTQTVLSKDPNEKRDHMVL<br>HEYVNAAGITGSKKKKTAPTPPKRSGGS<br>DLNMPAYVKFNYMAEREDELSLIKGTK<br>VIVMEKSSDGWWRGSYNGQVGWFPSN<br>YVTEEGDSPLGSENLYFQGLEHHHHHH | |
| **GFP-SpyCatcher-6His** | MKGEELFTGVVPILVELDGDVNGHEFS<br>VRGEGEGDATIGKLTLKFICTTGKLPVP<br>WPTLVTTLTYGVQCFSRYPDHMKRHDF<br>FKSAMPEGYVQERTISFKDDGKYKTRA<br>VVKFEGDTLVNRIELKGTDFKEDGNILG<br>HKLEYNFNSHDVYITADKQENGIKAEFT<br>VRHNVEDGSVQLADHYQQNTPIGDGPV<br>LLPDNHYLSTQTVLSKDPNEKRDHMVL<br>HEYVNAAGITGSGAMVDTLSGLSSEQG<br>QSGDMTIEEDSATHIKFSKRDEDGKELA<br>GATMELRDSSGKTISTWISDGQVKDFYL<br>YPGKYTFVETAAPDGYEVATAITFTVNE<br>QGQVTVNGKATKGDAHIENLYFQGLEH<br>HHHHH | SpyCatcher: Streptococcus pyogenes fibronectin binding protein Fbab-B (PDB:2x5p) residues -2-113[7] |
| **mCherry-PRM-SH3-6His** | MVSKGEEDNMAIIKEFMRFKVHMEGSV<br>NGHEFEIEGEGEGRPYEGTQTAKLKVTK<br>GGPLPFAWDILSPQFMYGSKAYVKHPAD<br>IPDYLKLSFPEGFKWERVMNFEDGGVV<br>TVTQDSSLQDGEFIYKVKLRGTNFPSDG<br>PVMQKKTMGWEASSERMYPEDGALKG<br>EIKQRLKLKDGGHYDAEVKTTYKAKKP<br>VQLPGAYNVNIKLDITSHNEDYTIVEQY<br>ERAEGRHSTGGMDELYKGGSKKKKTAP<br>TPPKRSGGSDLNMPAYVKFNYMAERED<br>ELSLIKGTKVIVMEKSSDGWWRGSYNG<br>QVGWFPSNYVTEEGDSPLLEHHHHHH | For cell experiments |
| **mCherry-PRM(H)-SH3-6His** | MVSKGEEDNMAIIKEFMRFKVHMEGSV<br>NGHEFEIEGEGEGRPYEGTQTAKLKVTK<br>GGPLPFAWDILSPQFMYGSKAYVKHPAD<br>IPDYLKLSFPEGFKWERVMNFEDGGVV<br>TVTQDSSLQDGEFIYKVKLRGTNFPSDG<br>PVMQKKTMGWEASSERMYPEDGALKG<br>EIKQRLKLKDGGHYDAEVKTTYKAKKP<br>VQLPGAYNVNIKLDITSHNEDYTIVEQY<br>ERAEGRHSTGGMDELYKGGSKKTPPPV<br>PPRTTSKGGSDLNMPAYVKFNYMAERE<br>DELSLIKGTKVIVMEKSSDGWWRGSYN<br>GQVGWFPSNYVTEEGDSPLLEHHHHHH | For cell experiments |

## References


1. Greenfield, N.J. Using circular dichroism collected as a function of temperature to determine the thermodynamics of protein unfolding and binding interactions. *Nat. Protoc.* **1**, 2527–2535 (2006).
2. Li, P. et al. Phase transitions in the assembly of multivalent signalling proteins. *Nature* **483**, 336-340 (2012).
3. Banani, S.F. et al. Compositional Control of Phase-Separated Cellular Bodies. *Cell* **166**, 651-663 (2016).
4. Hoelz, A. et al. Crystal structure of the SH3 domain of betaPIX in complex with a high affinity peptide from PAK2. *J. Mol. Biol.* **358**, 509-522 (2006).
5. Gorelik, M. & Davidson, A.R. Distinct peptide binding specificities of Src homology 3 (SH3) protein domains can be determined by modulation of local energetics across the binding interface. *J. Biol. Chem.* **287**, 9168-9177 (2012).
6. Kim, Y.E., Kim, Y.N., Kim, J.A., Kim, H.M. & Jung, Y. Green fluorescent protein nanopolygons as monodisperse supramolecular assemblies of functional proteins with defined valency. *Nat. Commun.* **6**, 7134 (2015).
7. Zakeri, B. et al. Peptide tag forming a rapid covalent bond to a protein, through engineering a bacterial adhesin. *Proc. Natl. Acad. Sci. USA* **109**, E690-E697 (2012).
